# Effect of level of sedation on outcomes in critically ill adult patients: a systematic review of clinical trials with meta-analysis and trial sequential analysis

**DOI:** 10.1016/j.eclinm.2024.102569

**Published:** 2024-03-28

**Authors:** Ameldina Ceric, Johan Holgersson, Teresa L. May, Markus B. Skrifvars, Johanna Hästbacka, Manoj Saxena, Anders Aneman, Anthony Delaney, Michael C. Reade, Candice Delcourt, Janus Christian Jakobsen, Niklas Nielsen

**Affiliations:** a Anesthesia & Intensive Care, Department of Clinical Sciences, Lund University, Skane University Hospital, Malmö, Sweden; b Lund University, Helsingborg Hospital, Department of Clinical Sciences Lund, Anesthesia & Intensive Care, Lund, Sweden; c Maine Medical Center, Department of Critical Care, Portland, Maine, USA; d Department of Emergency Care and Services, Helsinki University Hospital and University of Helsinki, Helsinki, Finland; e Department of Anesthesiology and Intensive Care, Tampere University Hospital and Tampere University, Tampere, Finland; f Division of Critical Care, George Institute for Global Health, Australia; g St. George Hospital, South Eastern Sydney Local Health District, Sydney, Australia; h Intensive Care Unit, Liverpool Hospital, South Western Sydney Local Health District, South Western Sydney Clinical School, University of New South Wales, Faculty of Medicine, Health and Human Sciences, Macquarie University, Sydney, Australia; i The George Institute for Global Health, Sydney, NSW, Australia; j Medical School, University of Queensland, Brisbane, QLD, Australia; k The George Institute for Global Health, Faculty of Medicine, University of New South Wales, Sydney, NSW, Australia; l Copenhagen Trial Unit – Centre for Clinical Intervention Research, Rigshospitalet, Copenhagen University Hospital, Copenhagen, Denmark; m Department of Regional Health Research, The Faculty of Health Sciences, University of Southern Denmark, Denmark; n Department of Clinical Medicine, Faculty of Medicine, Health and Human Sciences, Macquarie University, Sydney, NSW, Australia

**Keywords:** Systematic review, Meta-analysis, Critically ill, Intensive care, Sedation, Mortality

## Abstract

**Background:**

Sedation is routinely administered to critically ill patients to alleviate anxiety, discomfort, and patient-ventilator asynchrony. However, it must be balanced against risks such as delirium and prolonged intensive care stays. This study aimed to investigate the effects of different levels of sedation in critically ill adults.

**Methods:**

Systematic review with meta-analysis and trial sequential analysis (TSA) of randomised clinical trials including critically ill adults admitted to the intensive care unit. CENTRAL, MEDLINE, Embase, LILACS, and Web of Science were searched from their inception to 13 June 2023. Risks of bias were assessed using the Cochrane risk of bias tool. Primary outcome was all-cause mortality. Aggregate data were synthesised with meta-analyses and TSA, and the certainty of the evidence was assessed using the Grading of Recommendations, Assessment, Development and Evaluation (GRADE) approach. This study is registered with PROSPERO: CRD42023386960.

**Findings:**

Fifteen trials randomising 4352 patients were included, of which 13 were assessed high risk of bias. Meta-analyses comparing lighter to deeper sedation showed no evidence of a difference in all-cause mortality (risk ratio (RR) 0.94, 95% confidence interval (CI) 0.83–1.06; p = 0.28; 15 trials; moderate certainty evidence), serious adverse events (RR 0.99, CI 0.92–1.06; p = 0.80; 15 trials; moderate certainty evidence), or delirium (RR 1.01, 95% CI 0.94–1.09; p = 0.78; 11 trials; moderate certainty evidence). TSA showed that when assessing mortality, a relative risk reduction of 16% or more between the compared interventions could be rejected.

**Interpretation:**

The level of sedation has not been shown to affect the risks of death, delirium, and other serious adverse events in critically ill adult patients. While TSA suggests that additional trials are unlikely to significantly change the conclusion of the meta-analyses, the certainty of evidence was moderate. This suggests a need for future high-quality studies with higher methodological rigor.

**Funding:**

None.

**Research in context**

**Evidence before this study**

In a preliminary search of PubMed, Web of Science, Embase, and Cochrane Library databases, spanning from inception to March 5th, 2024, for each database, we reviewed the existing evidence on the effect of sedation on critically ill adult patients. We used specific search terms “sedation OR hypnotics” AND “critically ill OR critical care OR intensive care” AND “adult” AND “meta-analyses”. A systematic review and meta-analyses published in 2020 investigated the effect of light sedation compared to deep sedation in critically ill adults and found that the deeper sedation group had a significantly increased risk for death. In contrast, a meta-analysis published in 2021 showed in meta-analysis of the included randomised trials showed no evidence of a difference in intensive care mortality. Furthermore, a meta-analysis published in 2018 showed lower mortality rate in patients treated with lighter sedation compared with deeper sedation.

**Added value of this study**

Thus, the previously conducted meta-analyses are inconclusive and this study addresses the limitations of prior meta-analyses by considering the risks of both systematic errors and random errors including Trial Sequential Analysis (TSA), that may enhance the robustness of our analysis and provide a more comprehensive evaluation of the available evidence.

**Implications of all the available evidence**

This meta-analysis suggests that the level of sedation does not seem to affect the risks of death, serious adverse events, or delirium in critically ill adult patients. While the TSA indicates that additional trials are unlikely to significantly change these findings, the moderate certainty of evidence and the high risk of bias in the included studies highlights the importance for future high-quality trials with increased methodological rigour to ensure more reliable conclusions.

## Introduction

Patients with acute serious illnesses who require intensive care admission, also require effective treatment of associated discomfort, anxiety, agitation, and pain that occurs during the process of resuscitation, diagnostics, and subsequent management. The patient's ability to communicate discomfort and pain is often compromised by the several factors including severity of illness, altered mental status, medications, and the need for organ support.^1^ Clinical status changes frequently, so clinicians need to continuously assess patient symptoms to assure appropriate titration of sedatives and analgesics.^2^^,^^3^

In the short-term, sedatives are primarily used to combat anxiety, agitation, and to prevent patient-ventilator asynchrony. They also decrease the level of consciousness and reduce the capacity of the patient to respond to stimuli and interact with the environment. Sedatives blunt the sympathetic response and may cause cardiovascular dysfunction. In the medium-to long-term, deep sedation is associated with prolonged length of intensive care unit (ICU) stay and associated complications such as delirium.^4^^,^^5^ Post-traumatic stress disorder is common after acute serious illness and may be related to sedative use or choice of sedative agent.^6^, ^7^, ^8^ By tailoring sedation to the patients' needs and circumstances, health care providers can determine appropriate level of sedation and manage adverse events. This can be achieved by considering patient-related factors such as age, gender, past medical history, the trajectory of the illness, and the pharmacological properties of the agents used.^1^^,^^5^

In addition to these considerations, there has been a long-standing discussion about the potential risks and benefits of minimising the depth of sedation, particularly in the general, non-brain-injured ICU population.^1^^,^^5^^,^^9^^,^^10^ In patients with brain injury, there is an additional need to manage increased intracranial pressure and seizures and closely monitor the patient's response to stimuli. In practice clinicians often use sedation scales to assess the effect of sedatives on anxiety, agitation, and level of consciousness, to alter the dose of sedatives and target a level of sedation.^5^^,^^10^ Observational trials have shown a correlation between deeper sedation and adverse outcomes, including mortality and duration of mechanical ventilation.^11^^,^^12^ However, these studies possess inherent limitations, notably incomplete adjustment for illness severity. For instance, participants who are eligible for lighter sedation are those who are least likely to have poor outcomes. Adjusting for this confounding factor using observed indices of illness severity presents challenges. Randomised clinical trials are the most effective approach to address this confounding. Despite several randomised clinical trials addressing the question, the balance of risk and benefit associated with light sedation is neither clear nor universally accepted in clinical practice. The previously conducted meta-analyses have some important limitations.^9^^,^^13^^,^^14^ This study addresses the limitations of prior meta-analyses by considering the risks of both systematic errors and random errors including Trial Sequential Analysis (TSA).^15^ TSA, a methodology not utilized in previous studies, may enhance the robustness of our analysis, and provide a more comprehensive evaluation of the available evidence. Accordingly, the primary aim of this study was to investigate the association of level of sedation with all-cause mortality by undertaking a quantitative assessment of all relevant published clinical trials. Secondary aims were to identify associations between level of sedation, neurological outcome, and serious adverse events. Our hypothesis was that lighter sedation compared with deeper sedation reduces the risk of death by 25% in critically ill adult patients admitted to the ICU.

## Methods

### Search strategy and selection criteria

This systematic review, incorporating meta-analyses and trial sequential analysis (TSA) of randomised clinical trials, was conducted in accordance with the PRISMA (Preferred Reporting Items for Systematic Reviews and Meta-Analyses) guideline. The PRISMA checklist was used to guide the reporting process and ensure the inclusion of essential items for a high-quality systematic review. The review protocol was registered prospectively on the international prospective registry of systematic reviews (CRD42023386960), and a pre-specified protocol was published.^16^ We searched all relevant databases (Cochrane Central Register of Controlled Trials (CENTRAL) in the Cochrane Library, MEDLINE, Embase, LILACS, Web of Science Core Collection) from their inception to 13 June 2023 and included randomised clinical trials including critically ill adults admitted to ICU. Trial inclusion required comparison of sedation with no sedation or lighter sedation (however defined by the included study) with deeper sedation. Studies comparing any intervention with one group targeting lighter sedation than the other group, were eligible for inclusion, irrespectively of methods (for example sedations scales, sedation protocol, or type of sedative drug) used to achieve this separation. Studies were not eligible if no separation of targeted sedation depth could be identified.

### Data analyses

The primary outcomes were all cause mortality at longest follow-up. Secondary outcomes were serious adverse events at any timepoint, poor neurological outcome (defined by trialists) at longest follow-up, and delirium at any time-point in the ICU admission. Exploratory outcomes were PTSD and duration of mechanical ventilation. In accordance with the Cochrane Handbook for Systematic Reviews of Interventions, two authors independently reviewed each trial for risk of bias, using the second version of the Cochrane risk of bias tool for randomised trials (RoB2).^17^ We calculated risk ratios with 95% confidence intervals (CI) by using meta-analyses for dichotomous outcomes. We performed meta-analyses by following the Cochrane Handbook of Systematic Reviews of Interventions, Keus and colleagues, and Jakobsen and colleagues.^18^ We used RStudio version 2022.02.3+492 to analyse the data. We combined a visual inspection of forest plots and statistical analyses to identify potential heterogeneity. We performed subgroup analyses (based on type of intervention, follow up time, and risk of bias) for the outcomes all-cause-mortality, serious adverse events and delirium to further investigate heterogeneity and to inspire hypotheses for future studies. Aiming to reduce the risk of type I and II errors, we used a multiplicity adjusted p-value and trial sequential analysis, by dividing the prespecified p value threshold with the value halfway between 1 (no adjustment) and the number of primary and secondary outcome comparisons (Bonferroni adjustment).^15^ Cumulative meta-analyses are at risk of random errors due to sparse data and multiple testing of accumulating data. Therefore, TSA can be applied to control these risks (http://www.ctu.dk/tsa/). Similar to a sample size calculation in a randomised clinical trial, TSA estimates the diversity-adjusted required information size (DARIS) (ie, the number of participants needed in a meta-analysis to detect or reject a certain intervention effect) in order to minimise random errors. Using TSA analyses, we pragmatically anticipated an intervention effect equal to a risk ratio reduction (RRR) of 25%, as recommended by the GRADE guidelines when previous evidence do not provide other preliminary estimations.^18^ Additionally, we used trial sequential analysis to define the lowest intervention-effects-threshold we can confirm or reject. We used the approach proposed by the Grading of Recommendations, Assessment, Development and Evaluation (GRADE) Working Group for rating the certainty of the evidence.^19^ A comprehensive description of the methods is provided in the Supplementary Materials and published protocol.^16^

### Ethics approval and consent to participate

No formal approval or review of ethics is required for this systematic review as individual patient data will not be included.

### Role of funding source

There was no funding source for this study. All authors had full access to all the data in the study and had final responsibility for the decision to submit for publication.

## Results

**Fig. 1:**
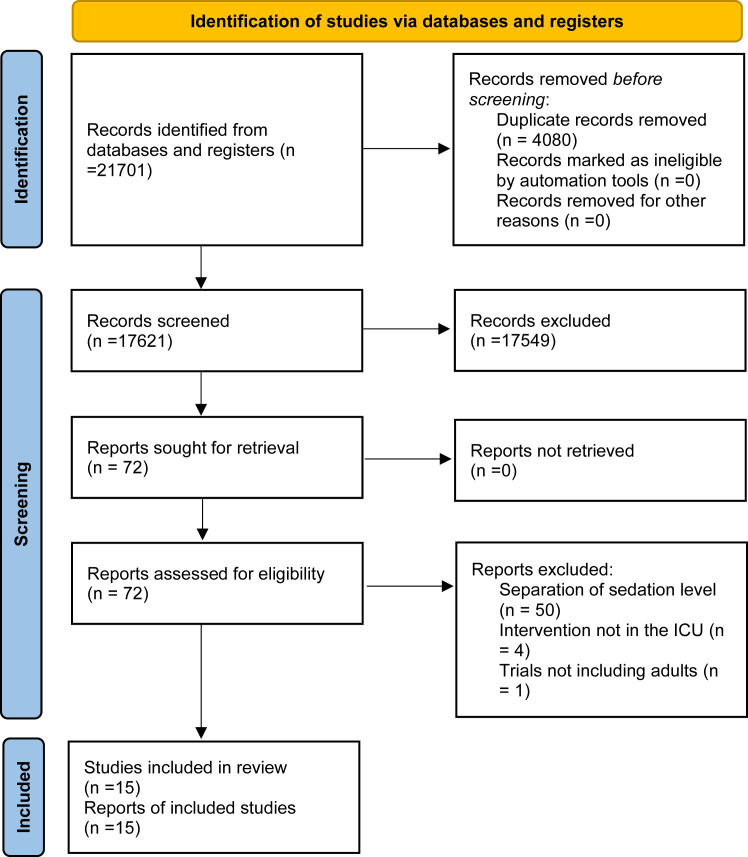
**PRISMA flow diagram outlining study inclusion**.

**Fig. 2:**
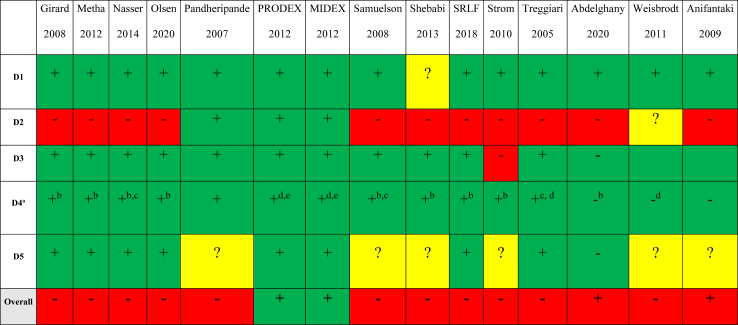
**Risk of bias summary**. Risk of bias summary for randomised controlled trials included in evidence. Synthesis. Risk of bias assessment used Cochrane risk of bias tool 2 (RoB2). D1, bias arising from randomisation process; D2, bias due to deviations from intended intervention; D3, bias due to missing outcome data; D4, bias in measurement of outcome; D5, bias in selection of reported result. +, low risk; ?, some concerns; -, high risk? ^a^ This is regarding all-cause mortality and serious adverse event as outcome. ^b^ High risk for outcomes delirium and duration of mechanical ventilation. ^c^ High risk for the outcome PTSD. ^d^ High risk of bias for the duration of mechanical ventilation outcome. ^e^ High risk for outcome delirium.

The search strategy defined in the protocol found 17,621 publications that were evaluated to identify trials matching our inclusion criteria. We included a total of 15 trials randomising 4352 participants (Fig. 1).^20^, ^21^, ^22^, ^23^, ^24^, ^25^, ^26^, ^27^, ^28^, ^29^, ^30^, ^31^, ^32^, ^33^ Funnel plot of included trials showed a symmetrical distribution around the effect estimate (risk ratio) suggesting minimal risk of publication bias (see Supplement Figure S1). Linear regression of the funnel plot (Egger's statistics) was not significant (intercept −0.1953, standard error = 0.56, p-value = 0.73) and this supported the visual inspection of the funnel plot that there are no clear signs of publication bias. Four trials with 2084 participants compared no sedation with sedation. Eleven trials involving 2268 participants were included to compare different sedation levels. Among these trials, four focused on comparing daily interruption of sedatives to continuous sedation, one trial compared intermittent sedation to daily interruption of sedatives, and two trials compared lighter sedation (defined as Motor activity assessment scale (MAAS) 3–4 or Modified Ramsey sedation scale level 1–2) to deeper sedation (defined as MAAS 1–2 or Modified Ramsey sedation scale level 3–4). Additionally, only four trials specified the type of sedative used, comparing dexmedetomidine to other sedatives. The characteristics of included studies and definition of the separation of sedation levels are presented in Supplement Table S1. Most participants (1843 in 12 trials) were hemodynamically unstable, and 1550 participants (10 trials) had respiratory failure. A minority of participants (150 participants in 7 trials) were trauma participants, 7 participants (1 trial) were neurologically injured participants, and no trials reported cardiac arrest participants. We assessed 13 trials as being of high risk of bias and 2 trials of being low risk of bias (Fig. 2). The most common reason for high risk of bias was the lack of successful blinding to treating clinicians which introduces potential bias through deviations from the intended intervention. The 15 included trials (Fig. 1) were included in meta-analyses. Missing data on the primary outcome constituted <5% of the overall data, and we deemed the impact of missing data to be low; therefore, we did not perform sensitivity analyses.

### Primary outcome

#### All-cause mortality

**Fig. 3:**
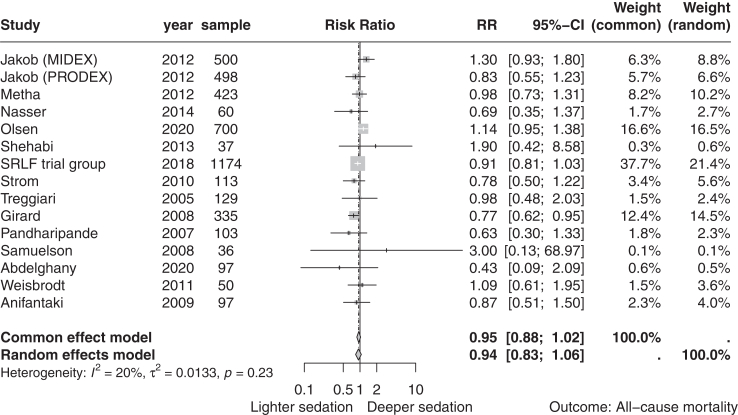
**Random effects meta-analysis comparing lighter sedation versus deeper sedation for all-cause mortality**. Random effects meta-analysis comparing lighter sedation versus deeper sedation for all-cause mortality (risk ratio 0.94, 95% confidence interval 0.83–1.06; p-val = 0.23; I^2^ = 20%; 15 trials). The risk ratios show a favor of lighter sedation to the left and deeper sedation to the right.

**Fig. 4:**
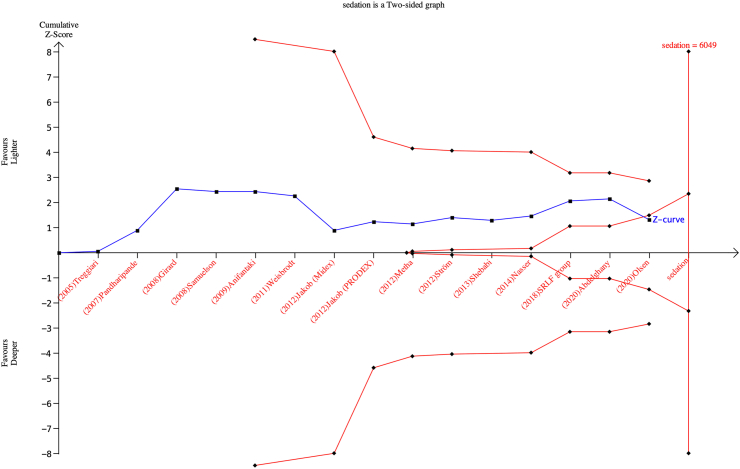
**Trial sequential analysis (TSA) of lighter sedation versus deeper sedation for all-cause mortality**. Two-sided TSA graph of lighter sedation versus deeper sedation for all-cause mortality in 15 trials. Diversity-adjusted required information size (DARIS) was calculated on basis of all-cause mortality proportion in control group of 36.4%, relative risk reduction of 16% in experimental group, type I error (α) of 2%, and type II error (β) of 10% (90% power). Required information size was calculated to be 7085 participants. Cumulative z curve (red lines above and under) did not cross trial sequential monitoring boundaries for either benefit or harm. Cumulative z curve did cross inner wedge futility line (red outward sloping lines).

**Fig. 5:**
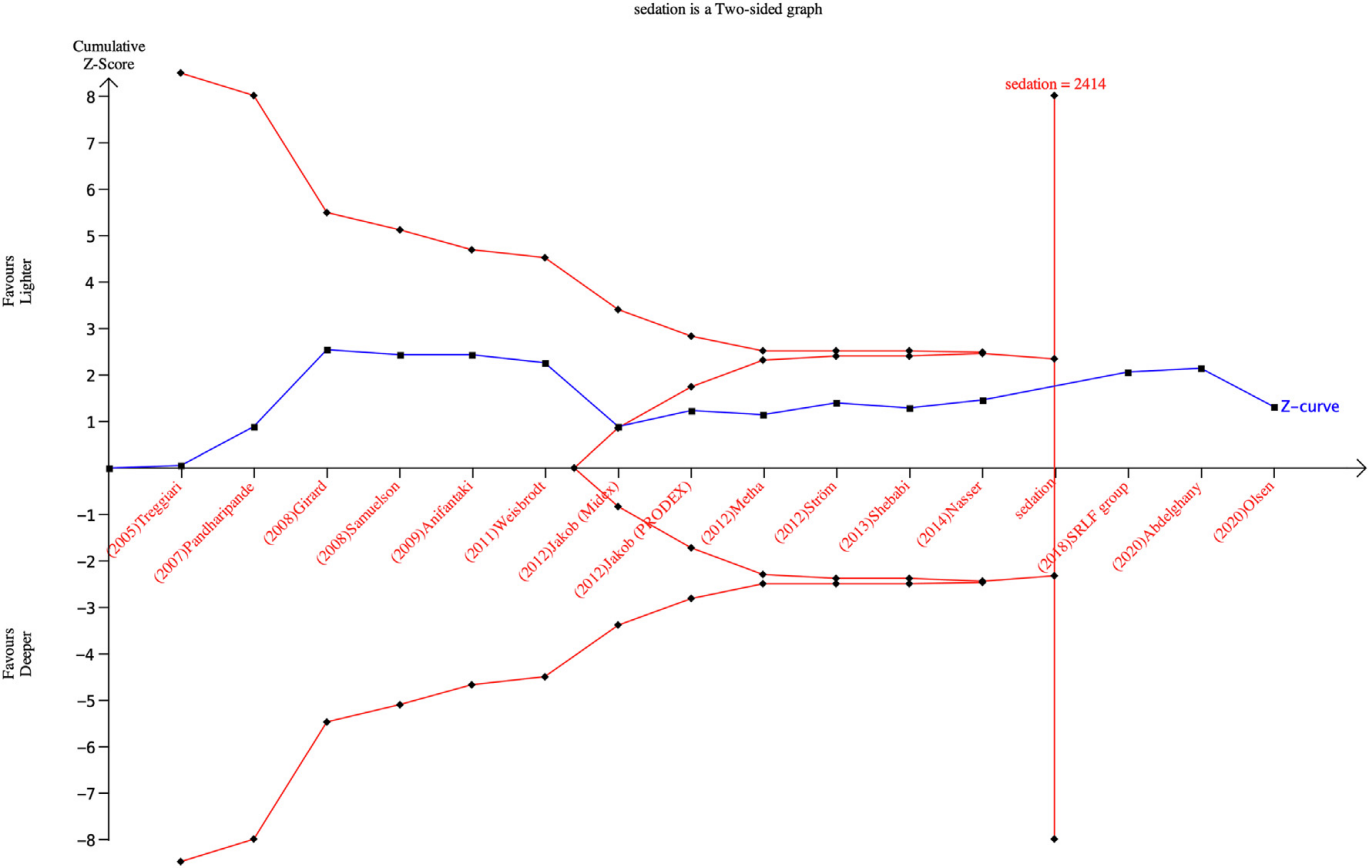
**Trial sequential analysis (TSA) of lighter sedation versus deeper sedation for all-cause mortality**. Two-sided TSA graph of lighter sedation versus deeper sedation for all-cause mortality in 15 trials. Diversity-adjusted required information size (DARIS) was calculated on basis of all-cause mortality proportion in control group of 36.4%, relative risk reduction of 25% in experimental group, type I error (α) of 2%, and type II error (β) of 10% (90% power). Required information size was calculated to be 2824 participants. Cumulative z curve (red lines above and under) did not cross trial sequential monitoring boundaries for either benefit or harm. Cumulative z curve did cross inner wedge futility line (red outward sloping lines).

Fifteen trials with a total of 4352 participants reported all-cause mortality. A total of 739 (33.9%) of 2177 in the lighter sedation group died compared to 748 (34.3%) of 2175 in the deeper sedation group. The timing of outcome assessment varied between trials, ranging from 28 days to 356 days after randomisation. Meta-analysis showed no evidence of a difference in all-cause mortality (risk ratio 0.94, 95% CI 0.83–1.06; I^2^ = 20%; p = 0.28; 15 trials; moderate certainty evidence) (Fig. 3; Table 1). Visual inspection of the forest plot and quantitative measures of heterogeneity (I^2^ = 20.0%) did not show clear signs of heterogeneity (Fig. 3). TSA showed that a relative risk reduction of 16% or more between the compared interventions could be rejected (Figs. 4 and 5). We assessed this outcome result as high risk of bias and the certainty of the evidence as moderate (Table 1).

**Table 1:** Summary of findings table for lighter sedation versus deeper sedation.

Lighter sedation compared to deeper sedation in critically ill adult patients.						
Patients or population: Critically ill adult patient admitted to intensive care unit. Setting: Admitted to intensive care unit. Intervention: Lighter sedation. Control: Deeper sedation.						
Outcome	Anticipated absolute effect size (95% CI)^c^		Relative effect size (95% CI)	No of participants (studies)	Certainty of evidence (GRADE)	Comments
	Risk with control	Risk with intervention				
All-cause mortality (follow up range: 28 days–365 days)	343 per 1000	339 per 1000	RR: 0.94 (0.83, 1.06)	4352 (15 RCT)	Moderate^a^	Risk of bias: Serious Inconsistency: No Indirectness: No Imprecision: No Publication bias: No
Serious adverse events (follow up range: 28 days–365 days)	411 per 1000	406 per 1000	RR: 0.99 (0.92, 1.06)	4352 (15 RCT)	Moderate^a^	Risk of bias: Serious Inconsistency: No Indirectness: No Imprecision: No Publication bias: No
Delirium (follow up range: 7 days–45 days)	332 per 1000	339 per 1000	RR: 1.01 (0.94, 1.09)	3368 (11 RCT)	Moderate^a^	Risk of bias: Serious Inconsistency: No Indirectness: No Imprecision: No Publication bias: No
PTSD (follow up range: 4 weeks-2 months)	88 per 1000	85 per 1000	RR: 0.97 (0.33, 2,85)	138 (2 RCT)	Low^a^^,^^b^	Risk of bias: Serious Inconsistency: No Indirectness: No Imprecision: yes Publication bias: No

RR: Risk ratio CI: Confidence interval; GRADE: GRADE Working Group grades of evidence.

GRADE Working Group grades of evidence.

High certainty: We are very confident that the true effect lies close to that of the estimate of the effect Moderate certainty: We are moderately confident in the effect estimate: The true effect is likely to be close to the estimate of the effect, but there is a possibility that it is substantially different Low certainty: Our confidence in the effect estimate is limited: The true effect may be substantially different from the estimate of the effect Very low certainty: We have very little confidence in the effect estimate: The true effect is likely to be substantially different from the estimate of effect.

Explanations:

a Downgraded one for risk of bias.

b Downgraded one for imprecision due to small sample size and wide confidence intervals.

c The risk in the intervention group (and its 95% confidence interval) is based on the assumed risk in the comparison group and the relative effect of the intervention (and its 95% CI).

### Secondary outcomes

#### Serious adverse events

Fifteen with a total of 4352 participants reported on serious adverse events. The most commonly reported serious adverse events (SAEs) included death (68.1% of all reported SAE) and secondary delirium (31.9% of all reported SAE). The assessment time points varied between trials, ranging from 28 days to hospital discharge, to 365 days after randomisation. A total of 883 (40.6%) of 2177 trial participants had a serious adverse event in the lighter sedation group compared with 893 (41.1%) of 2175 in the deeper sedation group. Meta-analysis showed no evidence of a difference (risk ratio 0.99, 0.92–1.06; I^2^ = 0%; p = 0.80; 15 trials; moderate certainty evidence) (Supplement Figure S2; Table 1). Quantitative assessment of heterogeneity (I^2^ = 0%) combined with visual inspection of the forest plot did not show signs of significant heterogeneity (Supplement Figure S2). TSA showed that a relative risk reduction of 9% or more between the compared interventions could be rejected (Supplement Figure S3 and S3a).

#### Neurological outcome

No trials reported on neurological outcome.

#### Delirium

Eleven trials with a total of 3368 participants reported on delirium. Eight trials used Confusion Assessment Method for the Intensive Care Unit (CAM-ICU), one used Diagnostic and Statistical Manual fourth edition (DSM-IV), and one used intensive care screening delirium checklist.^34^, ^35^, ^36^ The assessment time points varied between trials, ranging from 48 h, to ICU, to hospital discharge, to 28 days after randomisation. A total of 570 (33.9%) of 1681 trial participants had delirium in the lighter sedation group compared with 561 (33.2%) of 1687 in the deeper sedation group. Meta-analysis showed no evidence of a difference (risk ratio 1.01, 95% CI 0.94–1.09; p = 0.78; 11 trials; moderate certainty evidence) (Supplement Figure S4; Table 1). Quantitative measures of heterogeneity (I^2^ = 20%) combined with visual inspection of the forest plot did not show signs of significant heterogeneity (Supplement Figure S4). TSA showed that a relative risk reduction of 12% or more between the compared interventions could be rejected (Supplement Figure S5 and 5a).

### Exploratory outcomes

#### Duration of mechanical ventilation

Five trials including 1024 participants reported on the duration of mechanical ventilation. Meta-analyses showed no evidence of a difference (mean difference −0.91 (CI −2.01 to 0.18), p = 0.10; I^2^ = 0%; 5 trials) (Supplement Figure S6). Quantitative measures of heterogeneity (I^2^ = 0%) combined with visual inspection of the forest plot did not show signs of significant heterogeneity (Supplement Figure S6).

#### Posttraumatic stress disorder

One study (60 participants) reported higher median scores in the lighter sedation group using Impact of Event Scale, indicating higher psychological distress at 6 months follow up.^37^ Two studies (138 participants) used Impact of Event Scale Revised to report on post-traumatic stress disorder (PTSD) 2 months and 4 weeks follow up.^38^ Six (8.6%) out of 70 participants had PTSD in the lighter sedation group and 6 (8.8%) out of 68 participants had PTSD in the deeper sedation group. Meta-analysis showed no evidence of a difference (risk ratio 0.97, 95% CI 0.33–2.85; p = 0.95) (Supplement Figure S7). Quantitative measures of heterogeneity (I^2^ = 0%) combined with visual inspection of the forest plot did not show signs of significant heterogeneity (Supplement Figure S7).

#### Other exploratory outcomes

No studies reported data on quality of life, mean arterial blood pressure, body core temperature, or intracranial pressure.

### Subgroup analyses

None of the prespecified subgroup analyses showed evidence of a difference (Supplement Figures S8–S16).

## Discussion

In this systematic review with meta-analyses and trial sequential analysis of data from 15 randomised clinical trials and 4352 participants with moderate-level evidence, we showed that level of sedation did not seem to affect the risk of death in critically ill adults, based on studies conducted to 13 June 2023. We found almost no signs of statistical heterogeneity, and none of the predefined subgroup analyses showed evidence of a difference in all-cause mortality, which supports the validity of our meta-analysis results. We found no evidence that the level of sedation affected delirium or other serious adverse events. Further, we found no evidence that the level of sedation affected duration of mechanical ventilation or post-traumatic stress disorder. Finally, we found insufficient evidence to confirm or reject the hypothesis that the level of sedation affected neurological outcome. Among the fifteen included studies, thirteen were deemed to have a high risk of bias, primarily due to deviations from the intended intervention. The lack of blinding in the study designs extended to treating clinicians and outcome assessors, who were aware of trial participants' targeted sedation levels. This could have influenced medical decisions, potentially leading to adjustments in sedative dosages and other treatment approaches. Consequently, unintended deviations from the planned intervention might have affected patient outcomes, impacting factors such as recovery trajectories and clinical assessments. This could impact the validity and reliability of the study results, thus the overall level of evidence of these studies is moderate. The high risk of bias in these studies suggests a need for future studies with higher methodological rigor to address this limitation and provide more reliable results. It is difficult to blind the immediate treatment providers and patients to the allocated sedation level, however, other health care providers, outcome assessors, statisticians, and authors may be blinded to reduce the impact of not being able to blind the immediate treatment providers and patients.

The effects of different levels of sedation in critically ill patients remain uncertain, and consequently, the optimal assessment time point of mortality for such patients are not established. It is crucial to ensure that the duration of observation is sufficiently extended to allow physiological processes the necessary time to result in observable clinical events. However, the observation period must not extend too long, this might introduce events unrelated events to the intervention to occur, which might compromise the statistical power. Hence, for our primary analyses, we pragmatically selected the time to longest follow up a prior, adhering to this decision irrespectively of the study design or results, in accordance with the protocol.^16^

Our study included randomised clinical trials where it was possible to separate between different levels of sedation, regardless of the methodological approach used to define the targeted sedation level. The SPICE-III trial, comparing dexmedetomidine with usual sedation, aimed for “light sedation” using the RASS scale in both groups.^39^ As a result, there was no separation of targeted sedation levels, making it ineligible for inclusion in this review. Notably, despite a slightly higher proportion of patients with lighter RASS scores in the dexmedetomidine group (56.6% vs. 51.8%), no significant difference in outcomes was observed among the 4000 randomized patients. We included studies that used a protocolised approach to sedation, where one protocol aimed to achieve a lighter sedation level than the other group. The method used to achieve the targeted sedation level was not a criterion for study inclusion. Two studies used sedation scales (MAAS and modified Ramsey sedation scale) to differentiate between levels of sedation, but they used different types of sedation scales, and the approach was not consistent. It should be noted that using sedation scales to define levels of sedation is not inherently better or worse than other methods, and similarly, the protocolised sedation approach employed in three other studies (using daily interruption of sedatives or intermittent sedation as the lighter sedation group and continues as the deeper sedation group) is not necessarily superior. However, the difference in how the sedation scales were used in the two studies and the variation in the protocolised sedation approach used in these studies prevent direct comparisons of these results. Additionally, it should be noted that in some studies, the lighter sedation group may correspond to the deeper sedation group in other studies, making direct comparisons across studies classifying the groups into deeper versus lighter sedation even more challenging. For instance, two study used no sedation versus sedation with daily interruption, while three studies compared daily interruption with continuous sedation. The variation in sedation approaches, study design, and methods used to define sedation levels can make it challenging to interpret the results of these meta-analyses. The fact that different studies used different sedation protocols, sedation scales, or no sedation at all, means that the sedation levels achieved in the studies may not be directly comparable. Although our study did not find significant evidence of heterogeneity, it is essential to note that our results primarily demonstrate the effect of lighter sedation compared to deeper sedation aiming to achieve similar outcomes, regardless of the specific method used to achieve the targeted sedation level.

While the included studies used various methodologies to achieve the targeted sedation level, including sedation scales, protocolised sedation, interruption of sedatives, and no sedation; the results showed consistent effects of lighter sedation compared to deeper sedation. Although this variation in methodology may limit the reliability of the results, the fact that subgroups of these different methodologies also showed the same results increases the confidence in the overall findings. Nevertheless, the results should be interpreted cautiously, and further investigation and consideration of the various methodologies addressed in future studies. The lack of consensus on the definition of light, moderate, and deep sedation makes it challenging to evaluate the effect of the level of sedation on critically ill patients. Confounding factors, such as the severity of illness and underlying condition also affect the assessment of sedation depth. Therefore, investigating the effect of sedation is complex and warrants further high-quality studies to optimise care in critically ill adult patients.

TSA crossed the line of futility which adds to the robustness of our findings. Even though this suggests that additional trials are unlikely to change the conclusion of the meta-analyses significantly, it is essential to consider the moderate quality evidence included in the TSA, as this can impact the reliability and strength of the conclusion. Dealing with low-moderate quality evidence and high risk of bias can result in over- or underestimation of the true effect size. Therefore, when interpreting the TSA which are based on the effect estimate, it is important to consider the potential for bias and imprecision in the included trials. Thus, further research with similar methodologies used are unlikely to result in new findings, this study shows that future research must include refined methods and patient selection to determine if the level of sedation effects mortality. Specifically, future studies should aim to address the limitations of current evidence by using standardised methodology to assess the sedation depth and blinding of study participants and outcome assessors to reduce the risk of bias.

Our review has several strengths. Our method was predefined in detail, and the protocol was published before we performed our literature search. We searched all relevant databases, used an eight-step assessment suggested by Jakobsen and colleagues to assess our results' clinical significance, and we used TSA to reduce the risks of type I and type II errors.^15^ Furthermore, we did meta-analyses with both fixed effects and random effects meta-analysis, we investigated subgroup differences, and we assessed the certainty of the evidence through GRADE.

The main limitation of our review is the low-moderate methodological quality of the included trials, with most of the included trials were at high risk of bias. The inclusion of active comparator trials is a potentially complicating factor regarding interpretation of the results. The limitation of including active comparator trials (lighter versus deeper sedation, dexmedetomidine versus propofol, midazolam, and lorazepam, daily interruption versus continuous sedation) compared to intervention versus control (no sedation versus sedation) can complicate the interpretation of the results, as the results of active comparator trials can be influenced not only by the level of sedation but also by choice of sedatives and factors such as patient characteristics or clinical setting. Similarly, in trial comparing dexmedetomidine versus propofol, differences in pharmacological properties of the two drugs may impact the results, in addition to differences in the level of sedation. However, in the absence of heterogeneity between trials, as in our study, this should not be considered limiting to our results. Aiming to be inclusive, we accepted various patients and interventions. Furthermore, it is important to consider that the randomised clinical trials may have a potential weakness in this context, as they may not have included the sickest adult critically ill patients due lack of equipoise regarding lighter or deeper sedation. As a result, the trials may not have provided a comprehensive representation of the entire critically ill population. The inclusion of the most severely ill patients in the trials might have limited the ability to detect a mortality benefit associated with either lighter or deeper sedation strategies, leaving the question unanswered. Another limitation is in the secondary outcome delirium, where the assessment quality varies among studies, potentially impacting results. For instance, assessment frequency differs, ranging from one timepoint to daily assessments, and some studies lack detailed descriptions of the assessment methodology.

Guidelines suggest targeting lighter sedation or using daily awakening test to improve short-term outcomes, with low quality evidence.^1^^,^^40^ This study shows that lighter sedation compared to deeper sedation does not seem to affect mortality and other selected outcomes. However, it remains unknown whether this applies regardless the methods used to achieve the targeted sedation such as choice of sedative drug, choice of sedation scale used, or protocolised sedation. Our results suggest little to no difference in effect of the level of sedation, and we could reject a relative risk reduction of at least 16%. We acknowledge that a relative risk reduction of less than 16% may still be clinically relevant. Level of sedation may be investigated in further adequately powered high quality randomised trials, including a health economics perspective, to define implications for patients and society. Moreover, there is a notable paucity of studies specifically investigating the optimal level of sedation in patient populations such as brain injured patients and cardiac arrest patients, who pose unique challenges in sedation management. These critical patient groups, which often require intensive care management, are frequently excluded from randomised clinical trials assessing sedation strategies in critically ill patients. In particular, altered consciousness and neurological deficits in brain injured patients contribute to the complexity of accurately assessing and monitoring sedation levels, making using sedation scales difficult. Consequently, the generalisability of our findings to these relevant populations remains to be determined.

In summary, the level of sedation did not seem to affect the risks of death, serious adverse events, or delirium in critically ill adult patients. While the TSA suggests that additional trials are unlikely to significantly change the conclusion of the meta-analyses, the certainty of the evidence was only moderate. This suggests a need for future high-quality trials with increased methodological rigour to address this limitation and provide more reliable results.

## Contributors

AC, JCJ, and NN have substantially contributed with the concept and design of the work. AC has drafted the work and analysed the data. AC and JH accessed and verified the underlying data. All authors (AC, JHo, TLM, MBS, JHä, MS, AA, AD, MCR, CD, JCJ, and NN) had full access to the data, and have substantially contributed with interpreting the data and revised the work. All authors had final responsibility for the decision to submit for publication.

## Data sharing statement

All data generated or analysed during this study are included in this published article and its supplementary information files. Extracted data are available on request to the corresponding author.

## Declaration of interests

All authors have completed the ICMJE uniform disclosure form at www.icmje.org/coi_disclosure.pdf and declare: TLM declares that her institution received grants (ICECAP (UG3HL134269)) for her role as site principal investigator for SIREN funded RCT for cardiac arrest patients and are on clinical standardization committee. TLM's institution has secured funding for her involvement as a co-investigator in an ancillary study related to ICECAP, with a 5% Full-Time Equivalent support allocation (PRECICECAP (R01NS119825)). Additionally, TLM is principial investigator for a grant, reducing rural disparities in cardiac arrest outcomes by standardization of care (P20GM139745)), with 50% FTE support with funds to the institution. MSk received speakers fee 2021 and 2022 for BARD Medical (Ireland). JHä declares that she received grants from Paulo Foundation, Tor och Kirsti Johanssons Hjärt och Cancerstiftelse, Finska Läkaresällskapet, NordForsk, and Government funding for University Level research (2021, 2022, and 2023). JHä also declared that her institution (Tampere University Hospital research services) received a consultation fee from Paion and that JHä participated on Data Safety Monitoring Board or Adversary Board in Paion. JHä also received payment for lectures for Finnish Medical association, Laboratory Medicine, and Duodecim (a Finnish society of physicians). Additionally, JHä has a role in Educational Committee of Scandinavian Society of Anaesthesiology, board member of Advanced Educational Committee of Intensive Care Medicine in Scandinavian Society of Anaesthesiology and Intensive Care Medicine, and European Society of Intensive Care Medicine: National representative and faculty in CoBaTriCe Finnish Sepsis Society and have minor share of Orion B stock. MCR declare that his institution (University of Queensland) received grant support from National Health and Medical Research Council, Australia, Medical Research Future Fund, and Intensive Care Foundation, Australian Defence Force, and Royal Brisbane and Women's Hospital Foundation the past 36 months. MCR also received payment for expert testimony (in cases not related to the subject matter of this paper) from government of the Northern Territory High Court of New Zealand, received payment for being a member of DSMB for clod stored platelet trial, and his wife had stock investment in ETF that includes biomedical shares, which were sold 12 months ago. NN declared that his institution received support for the present study from Swedish Research Council and governmental funds within the Swedish Health Care (ALF). All other authors declared no conflicts of interests.

## References

[1] Devlin J.W., Skrobik Y., Gelinas C. (2018). Clinical practice guidelines for the prevention and management of pain, agitation/sedation, delirium, immobility, and sleep disruption in adult patients in the ICU. Crit Care Med.

[2] Vincent J.L., Shehabi Y., Walsh T.S. (2016). Comfort and patient-centred care without excessive sedation: the eCASH concept. Intensive Care Med.

[3] Chanques G., Pohlman A., Kress J.P. (2014). Psychometric comparison of three behavioural scales for the assessment of pain in critically ill patients unable to self-report. Crit Care.

[4] Celis-Rodríguez E., Díaz Cortés J.C., Cárdenas Bolívar Y.R. (2020). Evidence-based clinical practice guidelines for the management of sedoanalgesia and delirium in critically ill adult patients. Med Intensiva.

[5] DAS-Taskforce 2015, Baron R., Binder A. (2015). Evidence and consensus based guideline for the management of delirium, analgesia, and sedation in intensive care medicine. Revision 2015 (DAS-Guideline 2015) - short version. Ger Med Sci.

[6] Girard T.D., Shintani A.K., Jackson J.C. (2007). Risk factors for post-traumatic stress disorder symptoms following critical illness requiring mechanical ventilation: a prospective cohort study. Crit Care.

[7] Nassar Junior A., Zampieri F., Ranzani O., Park M. (2015). Protocolized sedation effect on post-ICU posttraumatic stress disorder prevalence: a systematic review and network meta-analysis. J Crit Care.

[8] Kress J.P., Gehlbach B., Lacy M., Pliskin N., Pohlman A.S., Hall J.B. (2003). The long-term psychological effects of daily sedative interruption on critically ill patients. Am J Respir Crit Care Med.

[9] Stephens R.J., Dettmer M.R., Roberts B.W. (2018). Practice patterns and outcomes associated with early sedation depth in mechanically ventilated patients: a systematic review and meta-analysis. Crit Care Med.

[10] Marra A., Ely E.W., Pandharipande P.P., Patel M.B. (2017). The ABCDEF bundle in critical care. Crit Care Clin.

[11] Shehabi Y., Chan L., Kadiman S. (2013). Sedation depth and long-term mortality in mechanically ventilated critically ill adults: a prospective longitudinal multicentre cohort study. Intensive Care Med.

[12] Balzer F., Weiß B., Kumpf O. (2015). Early deep sedation is associated with decreased in-hospital and two-year follow-up survival. Crit Care.

[13] Long L.R.S., Gong Y., Zhao H. (2020). Different depths of sedation versus risk of delirium in adult mechanically ventilated patients: a systematic review and meta-analysis. PLoS One.

[14] Aitken L.M., Kydonaki K., Blackwood B. (2021). Inconsistent relationship between depth of sedation and intensive care outcome: systematic review and meta-analysis. Thorax.

[15] Jakobsen J.C., Wetterslev J., Winkel P., Lange T., Gluud C. (2014). Thresholds for statistical and clinical significance in systematic reviews with meta-analytic methods. BMC Med Res Methodol.

[16] Ceric A., Holgersson J., May T. (2022). Level of sedation in critically ill adult patients: a protocol for a systematic review with meta-analysis and trial sequential analysis. BMJ Open.

[17] Higgins J.P.T., Savović J., Page M.J., Elbers R.G., Sterne J.A.C., Higgins J.P.T., Thomas J., Chandler J. (2022). Cochrane handbook for systematic reviews of interventions version 6.3 (updated February 2022).

[18] Higgins J.P.T., Thomas J., Chandler J. (2022). http://www.training.cochrane.org/handbook.

[19] Schünemann H.J., Higgins J.P.T., Vist G.E., Higgins J.P.T., Thomas J., Chandler J. (2022). Cochrane handbook for systematic reviews of interventions version 6.3 (updated February 2022).

[20] Treggiari M.M., Romand J.A., Yanez N.D. (2009). Randomized trial of light versus deep sedation on mental health after critical illness. Crit Care Med.

[21] Pandharipande P.P., Pun B.T., Herr D.L. (2007). Effect of sedation with dexmedetomidine vs lorazepam on acute brain dysfunction in mechanically ventilated patients: the MENDS randomized controlled trial. JAMA.

[22] Girard T.D., Kress J.P., Fuchs B.D. (2008). Efficacy and safety of a paired sedation and ventilator weaning protocol for mechanically ventilated patients in intensive care (Awakening and Breathing Controlled trial): a randomised controlled trial. Lancet.

[23] Samuelson K.A., Lundberg D., Fridlund B. (2008). Light vs. heavy sedation during mechanical ventilation after oesophagectomy--a pilot experimental study focusing on memory. Acta Anaesthesiol Scand.

[24] Strøm T., Martinussen T., Toft P. (2010). A protocol of no sedation for critically ill patients receiving mechanical ventilation: a randomised trial. Lancet.

[25] Jakob S.M., Ruokonen E., Grounds R.M. (2012). Dexmedetomidine vs midazolam or propofol for sedation during prolonged mechanical ventilation: two randomized controlled trials. JAMA.

[26] Mehta S., Burry L., Cook D. (2012). Daily sedation interruption in mechanically ventilated critically ill patients cared for with a sedation protocol: a randomized controlled trial. JAMA.

[27] Shehabi Y., Bellomo R., Reade M.C. (2013). Early goal-directed sedation versus standard sedation in mechanically ventilated critically ill patients: a pilot study∗. Crit Care Med.

[28] Nassar Junior A.P., Park M. (2014). Daily sedative interruption versus intermittent sedation in mechanically ventilated critically ill patients: a randomized trial. Ann Intensive Care.

[29] SRLF Trial Group (2018). Impact of oversedation prevention in ventilated critically ill patients: a randomized trial-the AWARE study. Ann Intensive Care.

[30] Olsen H.T., Nedergaard H.K., Strom T. (2020). Nonsedation or light sedation in critically ill, mechanically ventilated patients. N Engl J Med.

[31] Weisbrodt L., McKinley S., Marshall A.P., Cole L., Seppelt I.M., Delaney A. (2011). Daily interruption of sedation in patients receiving mechanical ventilation. Am J Crit Care.

[32] Abdelghany M.F., Alkarn A.F., Khalaf M.G., Gadalla W.A., Kamel E.Z. (2020). No-sedation in mechanically ventilated chronic obstructive pulmonary disease patients; a randomized controlled trial. Minia J Med Res.

[33] Anifantaki S., Prinianakis G., Vitsaksaki E. (2009). Daily interruption of sedative infusions in an adult medical-surgical intensive care unit: randomized controlled trial. J Adv Nurs.

[34] Ely E.W., Margolin R., Francis J. (2001). Evaluation of delirium in critically ill patients: validation of the confusion assessment method for the intensive care unit (CAM-ICU). Crit Care Med.

[35] Association AP (2000).

[36] Bergeron N., Dubois M.J., Dumont M., Dial S., Skrobik Y. (2001). Intensive care screening checklist: evaluation of a new screening tool. Intensive Care Med.

[37] Horowitz M.M., Wilner N.B., Alvarez W.M. (1979). Impact of event scale: a measure of subjective stress. Psychosom Med.

[38] Wilson P. (1997).

[39] Shehabi Y., Howe B.D., Bellomo R. (2019). Early sedation with dexmedetomidine in critically ill patients. N Engl J Med.

[40] Graham N.D., Graham I.D., Vanderspank-Wright B. (2022). A systematic review and critical appraisal of guidelines and their recommendations for sedation interruptions in adult mechanically ventilated patients. Aust Crit Care.

