# Supplementary material for: Effect of level of sedation on outcomes in critically ill adult patients: a systematic review of clinical trials with meta-analysis and trial sequential analysis
Source: eClinicalMedicine. 2024 Mar 28;71:102569. doi: 10.1016/j.eclinm.2024.102569 (PMC10990717; doi:10.1016/j.eclinm.2024.102569)
Supplement: Supplementary Figures and Tables [file mmc1.pdf]

Effect of level of sedation on outcomes in critically ill adult patients: a systematic review of clinical trials with meta-analysis and trial sequential analysis

Supplementary materials

Ameldina Ceric[1], Johan Holgersson[1], Teresa L May[2], Markus B Skrifvars[3], Johanna Hästbacka[4], Manoj Saxena[5], Anders Aneman[6], Anthony Delaney[7], Michael C Reade[8], Candice Delcourt[9], Janus Jakobsen[10,11], Niklas Nielsen[1]

Appendix

Supplementary methods **3**
Search strategies 5
Supplementary results *9*
Supplement table 1 9
Supplement table 2 11
Supplement figure 1. 13
Supplement figure 2. 14
Supplement figure 3. 15
Supplement figure 3a 16
Supplement figure 4. 17
Supplement figure 5 18
Supplement figure 5a 19
Supplement figure 6. 20
Supplement figure 7. 21
Subgroup analyses *22*
Supplement figure 8. 22
Supplement figure 9. 23
Supplement figure 10. 24
Supplement figure 11. 25
Supplement figure 12. 26
Supplement figure 13. 27
Supplement figure 14. 28
Supplement figure 15 29
Supplement figure 16 30

Supplementary methods

CENTRAL, MEDLINE, Embase, LILACS, and Web of Science Core Collection were searched to identify relevant trials. Databases were searched from their inception to 13 June 2023. We included randomised clinical trials irrespectively of design, setting, blinding, publication status, language, publication year and reporting of outcomes. We included adult patients admitted to an ICU (as defined by trialists), irrespectively of sex and comorbidities. Trials had to compare (1) any degree of sedation (as defined by trialists) compared with no sedation or (2) light sedation (as defined by trialists) compared with deep sedation (as defined by trialists). Studies comparing any intervention with one group receiving lighter sedation than the other group, were eligible for inclusion. Studies were not eligible if no separation of sedation depth could be identified.

Primary outcomes

- All-cause mortality at longest follow-up (dichotomous outcome).

Secondary outcomes

- Serious adverse event at any time point (dichotomous outcome). We will define a serious adverse event as any untoward medical occurrence that resulted in death, was life-threatening, required hospitalisation or prolongation of existing hospitalisation, or resulted in persistent or significant disability. Expecting the reporting of serious adverse events to be very heterogeneous and not strictly according to the 'International Council for Harmonisation of Technical Requirements for Registration of Pharmaceuticals for Human Use-Good Clinical Practice' (ICH-GCP) recommendations in many trials, we will include the event as a serious adverse event if the trialists either: (1) use the term 'serious adverse event' but not refer to ICH-GCP or (2) report the proportion of participants with an event we consider fulfil the ICH-GCP definition. If several of such events are reported, we will choose the highest proportion reported in each trial.
- Poor neurological outcome at longest follow-up (dichotomous outcome) (as defined by trialists).
- Delirium at any point in the ICU admission (dichotomous outcome) (as defined by trialists).

Exploratory outcomes

- Duration of mechanical ventilation (continuous scale) (as defined by trialists).

- Quality of life (any valid continuous scale).
- Post-traumatic stress disorder (dichotomous outcome).
- Mean arterial blood pressure (continuous scale).
- Body core temperature (continuous scale).
- Intracranial pressure (continuous scale).
- Duration of delirium/proportion of time spent in delirium (continuous scale).

Search strategies

Searches performed 13 June 2023

Total number of records identified: **21701 records**

Number of duplicates excluded: **4080 records**

Number of records in final list: **17621 records**

Cochrane Central Register of Controlled Trials (2023, Issue 6) in the Cochrane Library (4053 hits)

#1	MeSH descriptor: [Critical Illness] explode all trees
#2	MeSH descriptor: [Critical Care] explode all trees
#3	MeSH descriptor: [Intensive Care Units] explode all trees
#4	((critical* and (care* or ill*)) or intensive care or ICU or respirat* or ventilat*)
#5	#1 or #2 or #3 or #4
#6	MeSH descriptor: [Conscious Sedation] explode all trees
#7	MeSH descriptor: [Deep Sedation] explode all trees
#8	MeSH descriptor: [Hypnotics and Sedatives] explode all trees
#9	MeSH descriptor: [Benzodiazepines] explode all trees
#10	MeSH descriptor: [Propofol] explode all trees
#11	MeSH descriptor: [Dexmedetomidine] explode all trees
#12	(sedati* or benzodiazepin* or midazolam or propofol or dexmedetomidin* or lorazepam)
#13	#6 or #7 or #8 or #9 or #10 or #11 or #12
#14	#5 and #13
#15	(adult* or aged or elder* or middle next age* or old next age):ti,ab
#16	#14 and #15

MEDLINE Ovid (1946 to 13 June 2023) (3440 hits)

1.	exp Critical Illness/
2.	exp Critical Care/
3.	exp Intensive Care Units/

4. ((critical* and (care* or ill*)) or intensive care or ICU or respirat* or ventilat*).mp. [mp=title, abstract, original title, name of substance word, subject heading word, floating sub-heading word, keyword heading word, organism supplementary concept word, protocol supplementary concept word, rare disease supplementary concept word, unique identifier, synonyms]

5. 1 or 2 or 3 or 4

6. exp Conscious Sedation/

7. exp Deep Sedation/

8. exp "Hypnotics and Sedatives"/

9. exp Benzodiazepines/

10. exp Propofol/

11. exp Dexmedetomidine/

12. (sedati* or benzodiazepin* or midazolam or propofol or dexmedetomidin* or lorazepam).mp. [mp=title, abstract, original title, name of substance word, subject heading word, floating sub-heading word, keyword heading word, organism supplementary concept word, protocol supplementary concept word, rare disease supplementary concept word, unique identifier, synonyms]

13. 6 or 7 or 8 or 9 or 10 or 11 or 12

14. 5 and 13

15. (randomized controlled trial or controlled clinical trial or retracted publication or retraction of publication).pt. or clinical trials as topic.sh. or trial.ti.

16. (random* or blind* or placebo*).mp. [mp=title, abstract, original title, name of substance word, subject heading word, floating sub-heading word, keyword heading word, organism supplementary concept word, protocol supplementary concept word, rare disease supplementary concept word, unique identifier, synonyms]

17. 14 and (15 or 16)

18. limit 17 to ("all adult (19 plus years)" or "young adult (19 to 24 years)" or "adult (19 to 44 years)" or "young adult and adult (19-24 and 19-44)" or "middle age (45 to 64 years)" or "middle aged (45 plus years)" or "all aged (65 and over)" or "aged (80 and over)")

Embase Ovid (1974 to 13 June 2023) (10609 hits)

1. exp critical illness/

2. exp critically ill patient/

3. exp intensive care/

4. exp intensive care unit/

5. ((critical* and (care* or ill*)) or intensive care or ICU or respirat* or ventilat*).mp. [mp=title, abstract, heading word, drug trade name, original title, device manufacturer, drug manufacturer, device trade name, keyword heading word, floating subheading word, candidate term word]

6. 1 or 2 or 3 or 4 or 5

7. exp sedation/

8. exp hypnotic sedative agent/

9. exp benzodiazepine derivative/

10. exp propofol/

11. (sedati* or benzodiazepin* or midazolam or propofol or dexmedetomidin* or lorazepam).mp. [mp=title, abstract, heading word, drug trade name, original title, device manufacturer, drug manufacturer, device trade name, keyword heading word, floating subheading word, candidate term word]

12. 7 or 8 or 9 or 10 or 11

13. 6 and 12

14. Randomized controlled trial/ or Controlled clinical trial/ or retracted article/ or (erratum or tombstone).pt. or trial.ti. or yes.nr.

15. (random* or blind* or placebo*).mp. [mp=title, abstract, heading word, drug trade name, original title, device manufacturer, drug manufacturer, device trade name, keyword heading word, floating subheading word, candidate term word]

16. 13 and (14 or 15)

17. limit 16 to (adult <18 to 64 years> or aged <65+ years>)

LILACS (VHL Regional Portal; 1982 to 13 June 2023) (1338 hits)

((mh:(critical illness OR c23.550.291.625 OR critical care OR e02.760.190 OR n02.421.585.190 OR intensive care units OR n02.278.388.493 OR vs3.002.001.001.005)) OR (((critical* AND (care* OR ill*)) OR intensive care OR

icu OR respirat* OR ventilat*))) AND ((mh:(conscious sedation OR e03.250 OR deep sedation OR e03.295 OR hypnotics AND sedatives OR d27.505.696.277.350 OR d27.505.954.427.210.350 OR benzodiazepines OR d03.633.100.079.080 OR propofol OR d02.455.426.559.389.657.773 OR dexmedetomidine OR d03.383.129.308.245)) OR ((sedati* OR benzodiazepin* OR midazolam OR propofol OR dexmedetomidin* OR lorazepam))) AND (db:("LILACS"))

Science Citation Index Expanded (1900 to 13 June 2023) and Conference Proceedings Citation Index – Science (1990 to 13 June 2023) (Web of Science) (2261 hits)

#7 #5 AND #6

#6 TI=(adult* or aged or elder* or middle next age* or old next age) or AB=(adult* or aged or elder* or middle next age* or old next age)

#5 #3 AND #4

#4 TI=(random* or blind* or placebo* or meta-analys* or trial*) OR TS=(random* or blind* or placebo* or meta-analys*)

#3 #2 AND #1

#2 TS=(sedati* or benzodiazepin* or midazolam or propofol or dexmedetomidin* or lorazepam)

#1 TS=((critical* and (care* or ill*)) or intensive care or ICU or respirat* or ventilat*)

Supplementary results

Supplement table 1

Characteristics of included trials.

Trial ID	Intervention	Control	Number of participants	Sex (female %) (I/C)	Age (I/C)	All-cause mortality (I/C)	Serious adverse events	Delirium	Duration of mechanical ventilation	Days without mechanical ventilation	Post-traumatic stress disorder
Girard et al. T	Daily interruption of sedation	Continuous sedation	336	46/49	60 (48-71)/64 (51/75)	74/167 / 97/168	127/167 / 119/168	127/167 / 119/168	P=0.02	N/A	N/A
Jakob et al. T	Dexmedetomidine	Midazolam	501	39/30	65 (55-74)/65 (55-74)	63/249 / 49/251	63/249 / 49/251	18/247 / 17/250	P=0.033	N/A	N/A
Jakob et al. T	Dexmedetomidine	Propofol	500	36/33	65 (51-75)/65 (51-74)	37/251 / 44/257	37/251 / 44/257	6/246 / 16/247	P=0.4088	N/A	N/A
Metha et al. S	Protocolised sedation and daliy interruption of sedation	Protocolised sedation	430	43/44	57 (46-70)/60 (49-70)	63/214 / 63/209	113/214 / 113/209	113/214 / 113/209	N/A	N/A	N/A
Nasser et al. A	Intermittent sedation	Daily interruption of sedatives	60	43/57	47 (33-58)/51 (46-59)	9/30 / 13/30	12/30 / 13/30	12/30 / 9/30	N/A	N/A	N/A
Olsen et al. H	No sedation	Continuous sedation	710	36/42	72 (63-80)/70 (63-78)	148/349 / 130/351	148/349 / 130/351	N/A	N/A	P=0.16	N/A
Pandharipande et al. P	Dexmedetomidine	Lorazepam	106	42/55	60 (49-65)/59 (45-67)	9/52 / 14/51	41/52 / 42/51	41/52 / 42/51	N/A	P=0.22	N/A
Samuleson et al. K	Light sedation (MAAS 3-4)	Deep sedation (MAAS 1-2)	36	17/22	66 (±9.5)/66 (±10.0)	1/18 / 0/18	1/18 / 2/18	0/18 / 0/18	P=0.89	N/A	1/18 / 1/18
Shebabi et al. Y	Dexmedetomidine	Any other sedatives	37	48/44	65 (±15)/62 (±17)	5/21 / 2/16	8/21 / 6/12	8/21 / 6/12	N/A	P=0.72	N/A

Srlf trial group et al.	No sedation	Continuous sedation	1179	35/34	66 (±13)/ 67 (±14)	267/584 / 296/590	267/584 / 296/590	230/584 / 232/590	N/A	N/A	N/A
Strom et al. T	No sedation	Daily intterruption of sedation	140	24/41	67 (54-74)/ 65 (54-74)	20/55 / 27/58	20/55 / 27/58	11/55 / 4/58	N/A	P=0.0191	N/A
Treggiari et al. M	Modified Ramsay sedation scale level 1-2	Modified Ramsay sedation scale level 3- 4	137	25/22	63 (±15) /60 (±16)	12/65 / 12/64	12/65 / 12/64	N/A	P=0.02	N/A	5/52 / 5/50
Abdelghany et al. M	No sedation	Daily interruption of sedatives titrated to (RASS) - 4 - -5	97	30/118	62 (±9) /63 (±8)	2/47 / 5/50	11/47 / 11/50	7/47 / 3/50	P=0.5	P=0.6	N/A
Weisbrodt et al. L	Daily interruption of sedatives	Continuous sedation	50	46/29	65 (± 6.6)/ 69 (±5.4)	13/26 / 11/24	13/26 / 11/24	N/A	N/A	P=0.66	N/A
Anifantaki et al. S	Daily interruption of sedatives	Continuous sedation	97	29/27	52 (±21) /56(±21)	16/49 / 18/48	16/49 / 18/48	N/A	N/A	N/A	N/A

Supplement table 2

Summary of serious adverse events in the included trials

			**Lighter sedation group**		**Deeper sedation group**	
**Trial**	**Intervention**	**Control**	**Number and type of serious adverse event**	**Proportion of participants with a serious adverse event**	**Number and type of serious adverse event**	**Proportion of participants with a serious adverse event**
Girard et al. T	Daily interruption of sedation	Continuous sedation	124 delirium	124/167	119 delirium	119/168
Jakob et al. T	Dexmedetomidine	Midazolam	63 deaths	63/249	49 deaths	49/251
Jakob et al. T	Dexmedetomidine	Propofol	37 deaths	37/251	44 deaths	44/247
Metha et al. S	Protocolised sedation and daliy interruption of sedation	Protocolised sedation	113 delirium	113/214	113 delirium	113/209
Nasser et al. A	Intermittent sedation	Daily interruption of sedatives	12 delirium	12/30	13 deaths	13/30
Olsen et al. H	No sedation	Continuous sedation	148 deaths	148/349	130 deaths	130/351
Pandharipande et al. P	Dexmedetomidine	Lorazepam	41 delirium	41/52	42 delirium	42/51
Samuleson et al. K	Light sedation (MAAS 3-4)	Deep sedation (MAAS 1-2)	1 major post-operative complication leading to reintubation and prolonged ICU stay	1/18	2 major post-operative complication leading to reintubation and prolonged ICU stay	2/18
Shebabi et al. Y	Dexmedetomidine	Any other sedatives	8 delirium	8/21	6 delirium	6/16
Srlf trial group et al.	No sedation	Continuous sedation	267 deaths	267/584	296 deaths	296/590
Strom et al. T	No sedation	Daily intterruption of sedation	20 deaths	20/55	27 deaths	27/58
Treggiari et al. M	Modified Ramsay sedation scale level 1-2	Modified Ramsay sedation scale level 3- 4	12 deaths	12/65	12 deaths	12/64
Abdelghany et al. M	No sedation	Daily interruption of sedatives titrated to (RASS) -4 - -5	11/47 ventilator associated pneumonia	2/47	11/50 ventilator associated pneumonia	5/50 deaths

Weisbrodt et al. L	Daily interruption of sedatives	Continuous sedation	13/26 deaths	13/26	11/24 deaths	11/24
Anifantaki et al. S	Daily interruption of sedatives	Continuous sedation	16/49 deaths	16/49	18/48 deaths	18/48

Supplement figure 1. Funnel plot of included studies.

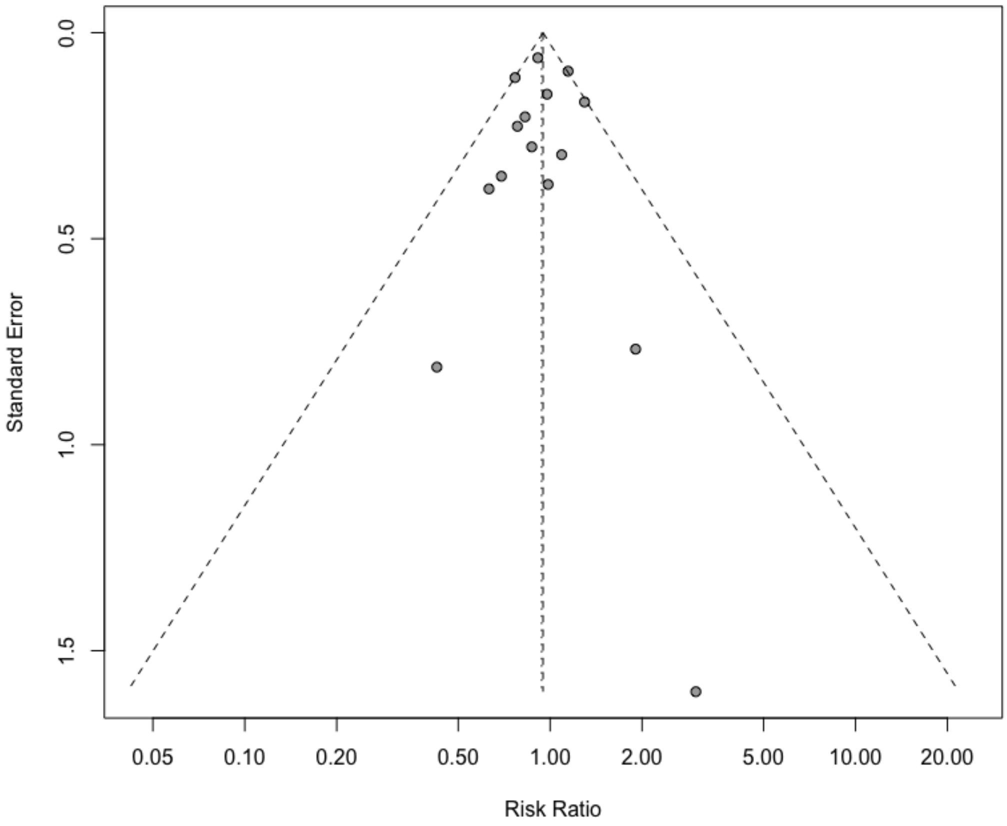

Funnel plot shows a symmetrical distribution around the effect estimate (risk ratio) suggestion minimal publication bias. Linear regression of the funnel plot (Egger's statistics) was not significant (intercept -0.1953, standard error = 0.56, p-value=0.73) and this supported the visual inspection of the funnel plot that there are no clear signs of publication bias.

Supplement figure 2.

Forest plot of serious adverse events for all studies.

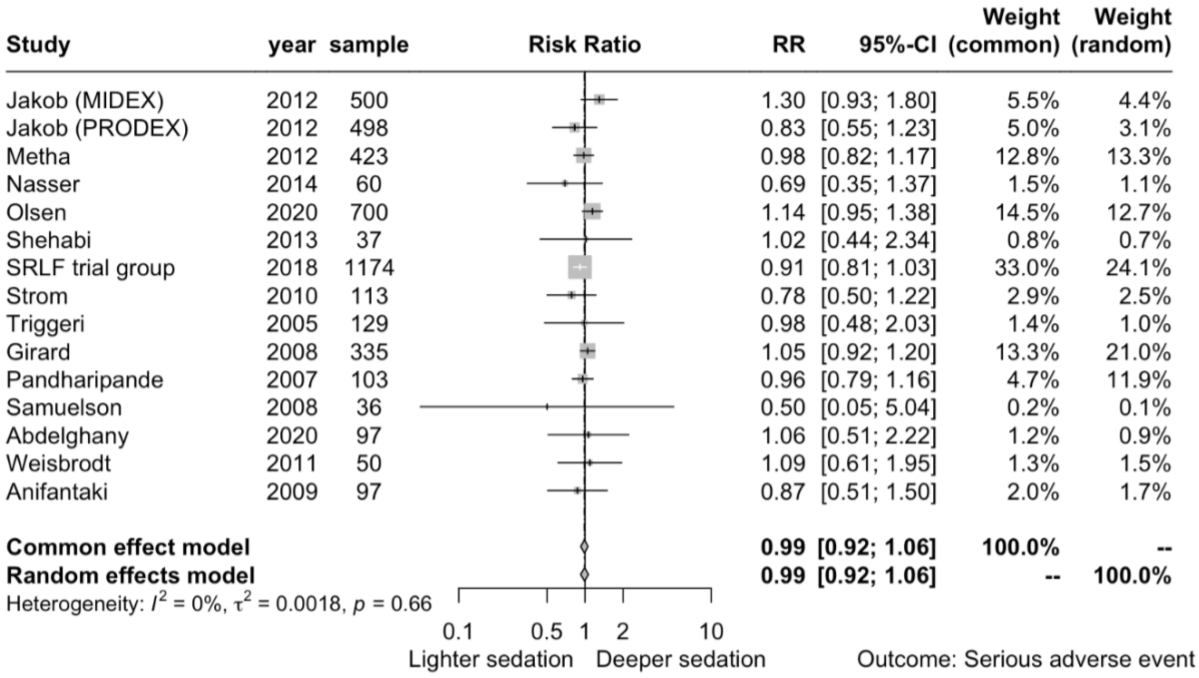

Supplement figure 2. Random effects meta-analysis comparing lighter sedation versus deeper sedation for serious adverse events (risk ratio 0.99, 95% confidence interval 0.92 to 1.06; p= 0.80; I^2=0%; 15 trials).

Supplement figure 3.

Trial sequential analyses (TSA) to define the lowest intervention-effects-threshold we can confirm or reject for serious adverse events (SAE).

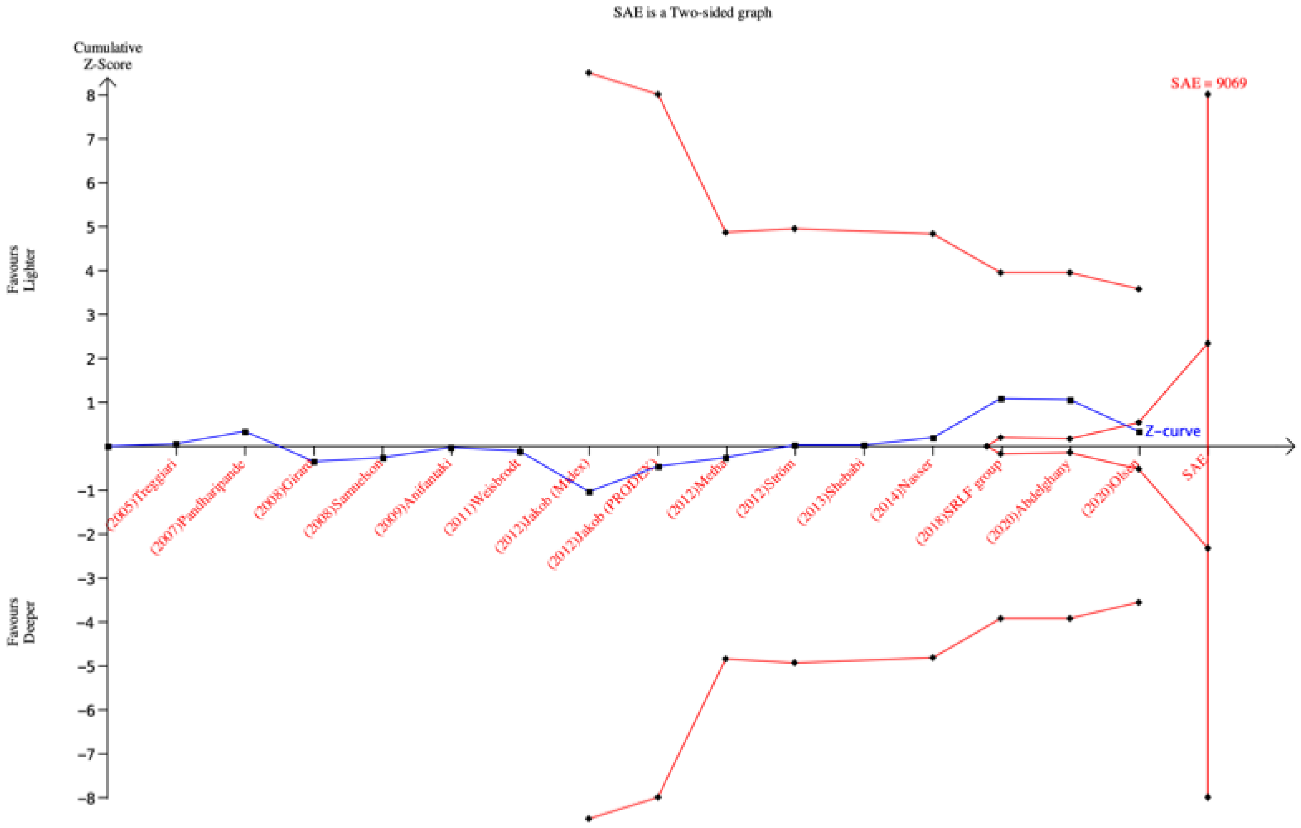

Supplement figure 3. Trial sequential analysis (TSA) of lighter sedation versus deeper sedation SAE. Two-sided TSA graph of lighter sedation versus deeper sedation for all-cause mortality in 15 trials. Diversity adjusted required information size (DARIS) was calculated on basis of SAE proportion in control group of 41.1%, relative risk reduction of 9 % in experimental group, type I error (α) of 2%, and type II error (β) of 10% (90% power). Required information size was calculated to be 9069 participants. Cumulative z curve (red lines above and under) did not cross trial sequential monitoring boundaries for either benefit or harm. Cumulative z curve did cross inner wedge futility line (red outward sloping lines).

Supplement figure 3a.

Trial sequential analyses for serious adverse events with relative risk reduction of 25%.

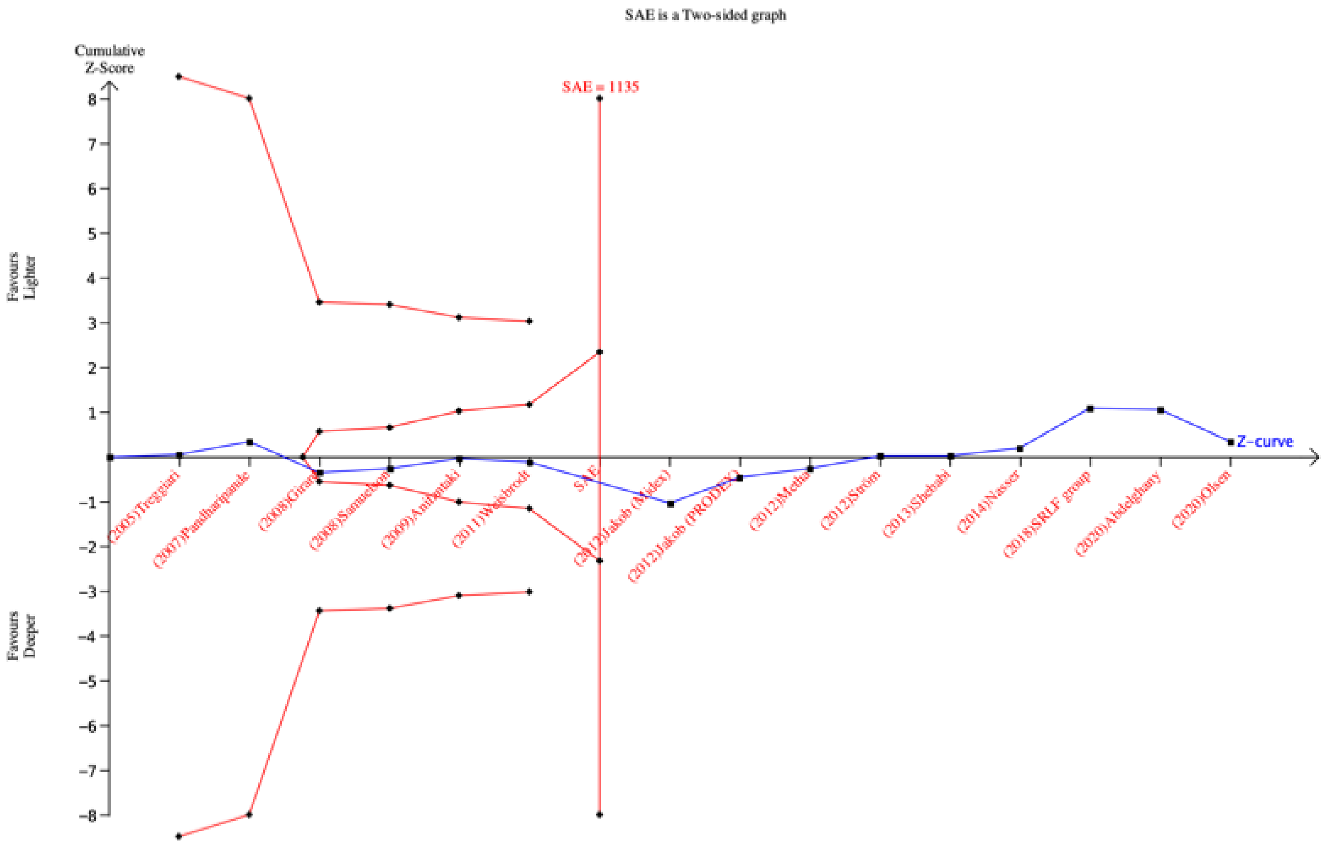

Supplement figure 3a. Trial sequential analysis (TSA) of lighter sedation versus deeper sedation SAE. Two-sided TSA graph of lighter sedation versus deeper sedation for all-cause mortality in 15 trials. Diversity adjusted required information size (DARIS) was calculated on basis of SAE proportion in control group of 41.6%, relative risk reduction of 25% in experimental group, type I error (α) of 2%, and type II error (β) of 10% (90% power). Required information size was calculated to be 1135 participants. Cumulative z curve (red lines above and under) did not cross trial sequential monitoring boundaries for either benefit or harm. Cumulative z curve did cross inner wedge futility line (red outward sloping lines).

Supplement figure 4.

Forest plot of delirium for all studies.

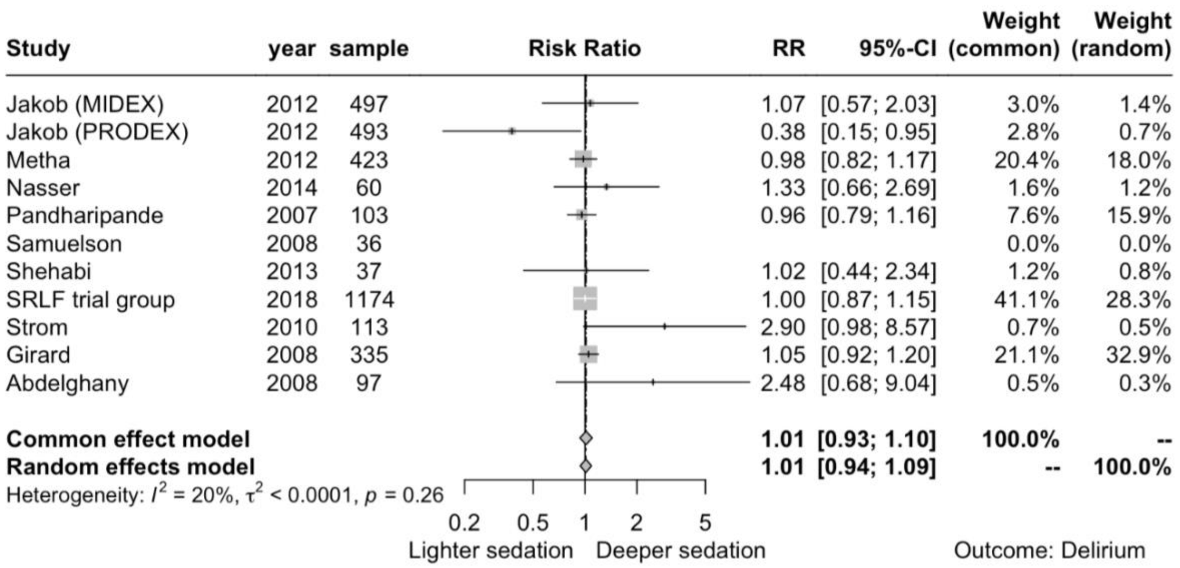

Supplement figure 4. Random effects meta-analysis comparing lighter sedation versus deeper sedation for delirium (risk ratio 1.01 favoring deeper sedation, 95% confidence interval 0.94 to 1.09; p= 0.78; I^2=20%; 11 trials). The risk ratios show a favor of lighter sedation to the left and deeper sedation to the right.

Supplement figure 5.

Trial sequential analyses to define the lowest intervention-effects-threshold we can confirm or reject for delirium.

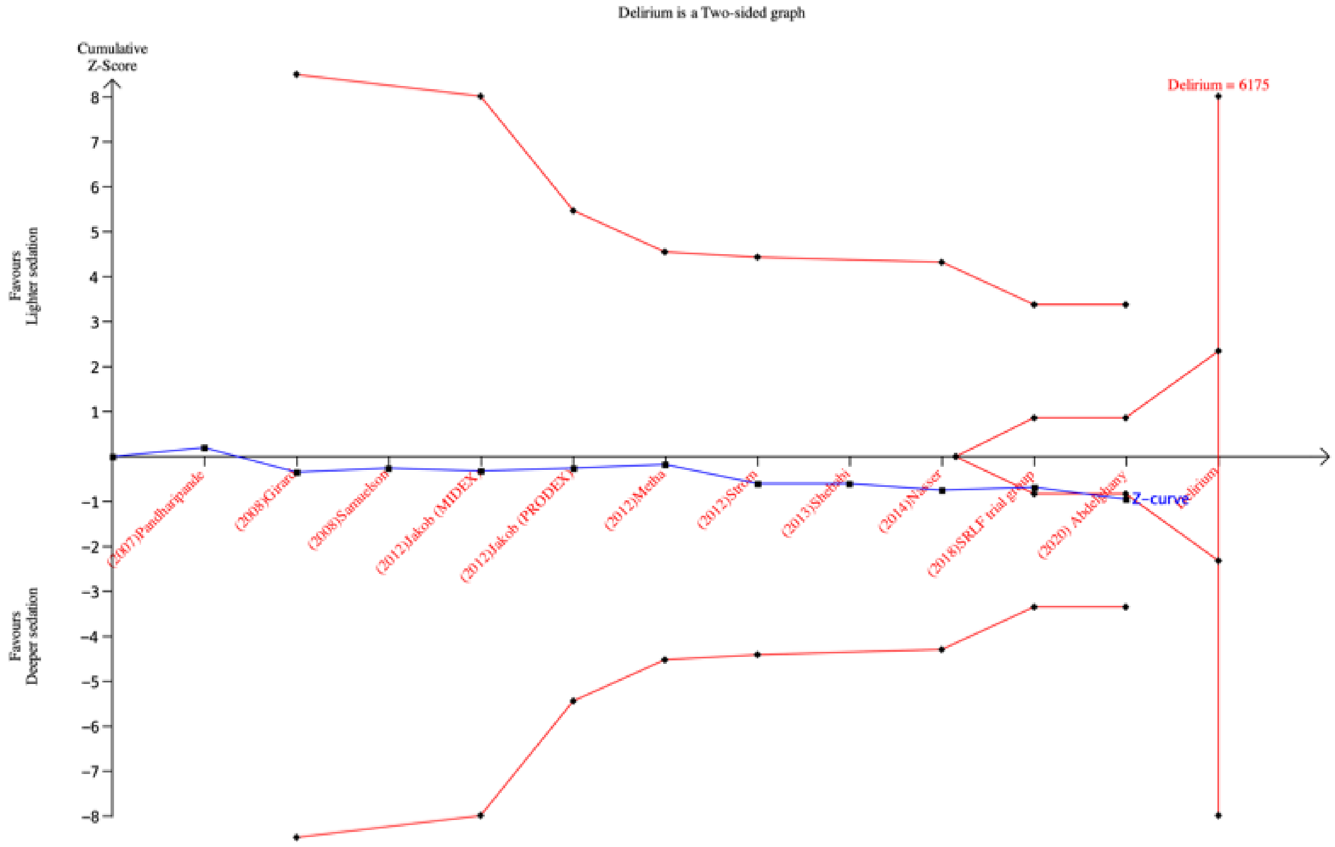

Supplement figure 5. Trial sequential analysis (TSA) of lighter sedation versus deeper sedation delirium. Two-sided TSA graph of lighter sedation versus deeper sedation for delirium in 11 trials. Diversity adjusted required information size (DARIS) was calculated on basis of delirium proportion in control group of 33.2%, relative risk reduction of 12% in experimental group, type I error (α) of 2%, and type II error (β) of 10% (90% power). Required information size was calculated to be 6175 participants. Cumulative z curve (red lines above and under) did not cross trial sequential monitoring boundaries for either benefit or harm. Cumulative z curve did cross inner wedge futility line (red outward sloping lines).

Supplement figure 5a.

Trial sequential analyses for delirium with relative risk reduction of 25%.

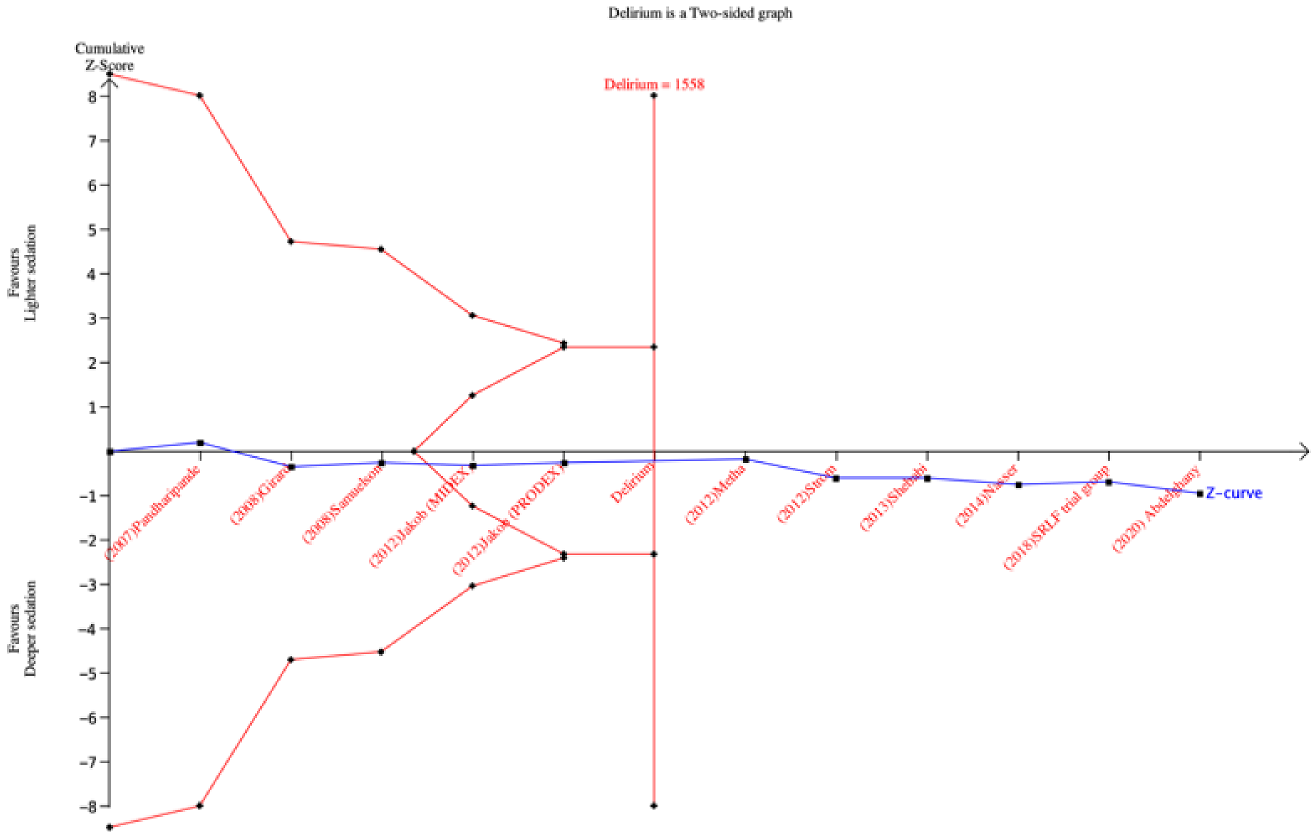

Supplement figure 5a. Trial sequential analysis (TSA) of lighter sedation versus deeper sedation delirium. Two-sided TSA graph of lighter sedation versus deeper sedation for delirium in 11 trials. Diversity adjusted required information size (DARIS) was calculated on basis of delirium proportion in control group of 33.2%, relative risk reduction of 25% in experimental group, type I error (α) of 2%, and type II error (β) of 10% (90% power). Required information size was calculated to be 1558 participants. Cumulative z curve (red lines above and under) did not cross trial sequential monitoring boundaries for either benefit or harm. Cumulative z curve did cross inner wedge futility line (red outward sloping lines).

Supplement figure 6.

Forest plot of duration of mechanical ventilation for all studies.

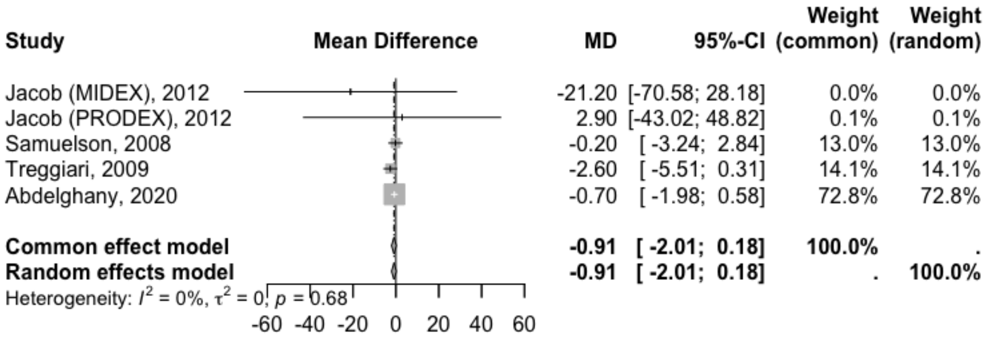

Supplement figure 6. Random effects meta-analysis comparing lighter sedation versus deeper sedation for duration of mechanical ventilation showed no evidence of a difference (mean difference -0.91 (CI -2.01 to 0.18), p=0.10; I^2=0%; 5 trials).

Supplement figure 7.

Forest plot for post-traumatic stress syndrome (PTSD) of all studies.

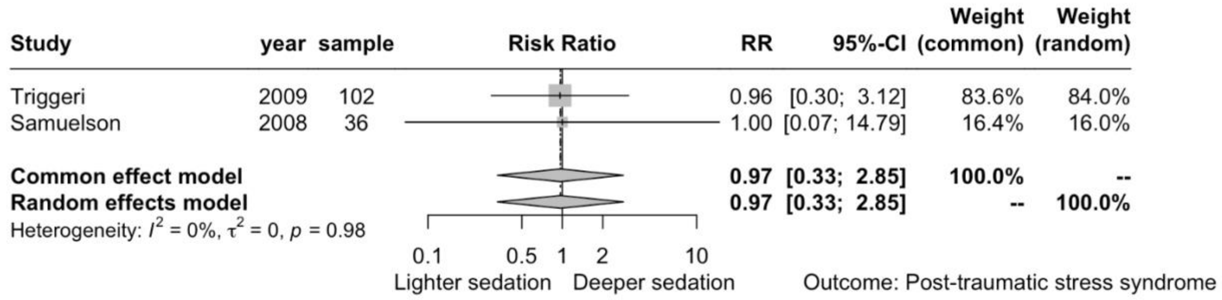

Supplement figure 7. Random effects meta-analysis comparing lighter sedation versus deeper sedation for PTSD (risk ratio 0.97, 95% confidence interval 0.33 to 2.85; p= 0.95; I^2=0%; 2 trials).

Subgroup analyses

Supplement figure 8.
Forest plot for all-cause mortality for all studies in subgroups by intervention type.

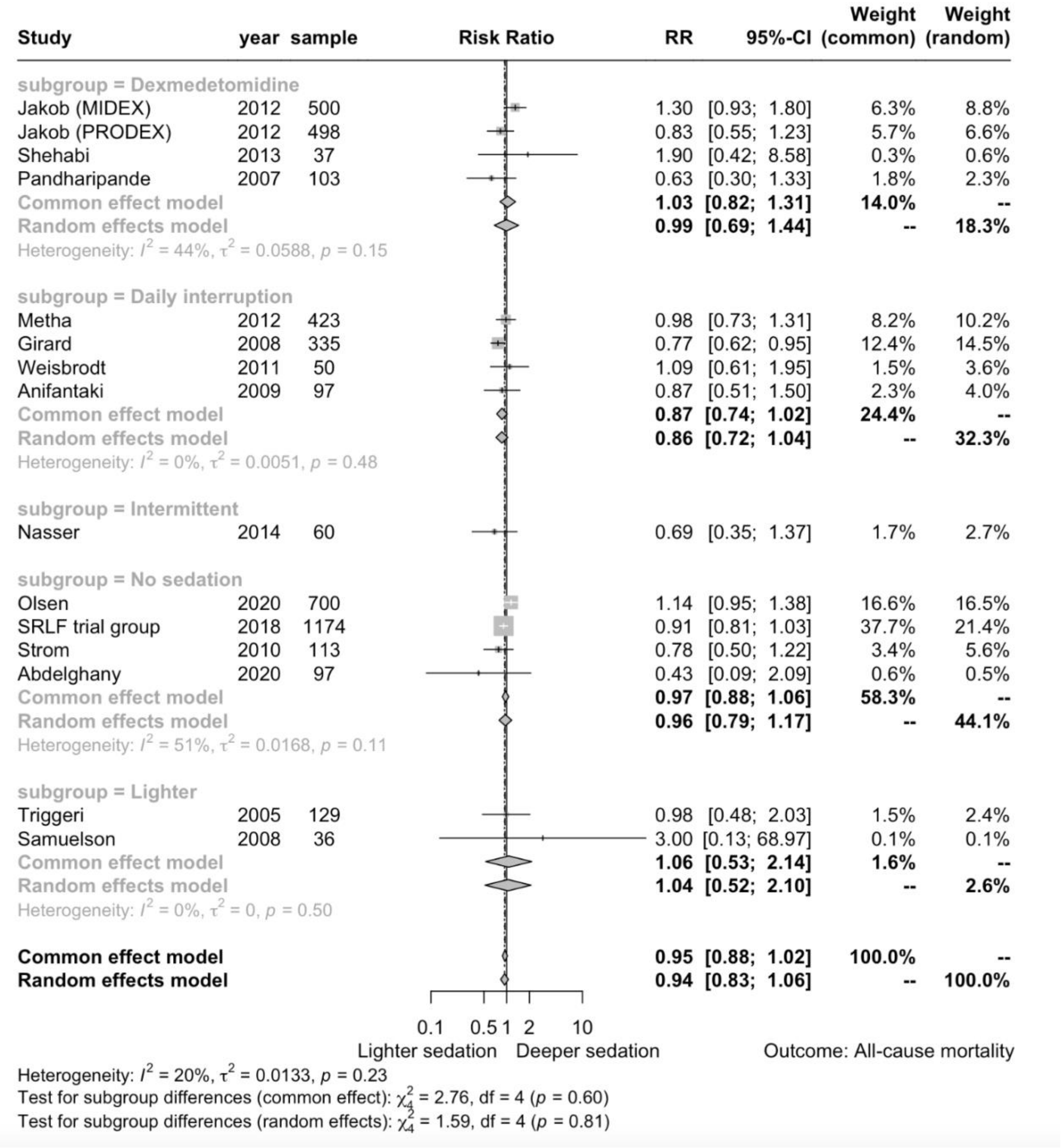

Random effects meta-analysis comparing lighter sedation versus deeper sedation for all-cause mortality with subgroups based on the intervention (risk ratio 0.94, 95% confidence interval 0.82 to 1.07; p-val=0.28; I^2=32%; 15 trials).

Supplement figure 9.

Forest plot for all-cause mortality for all studies in subgroups by time to follow-up.

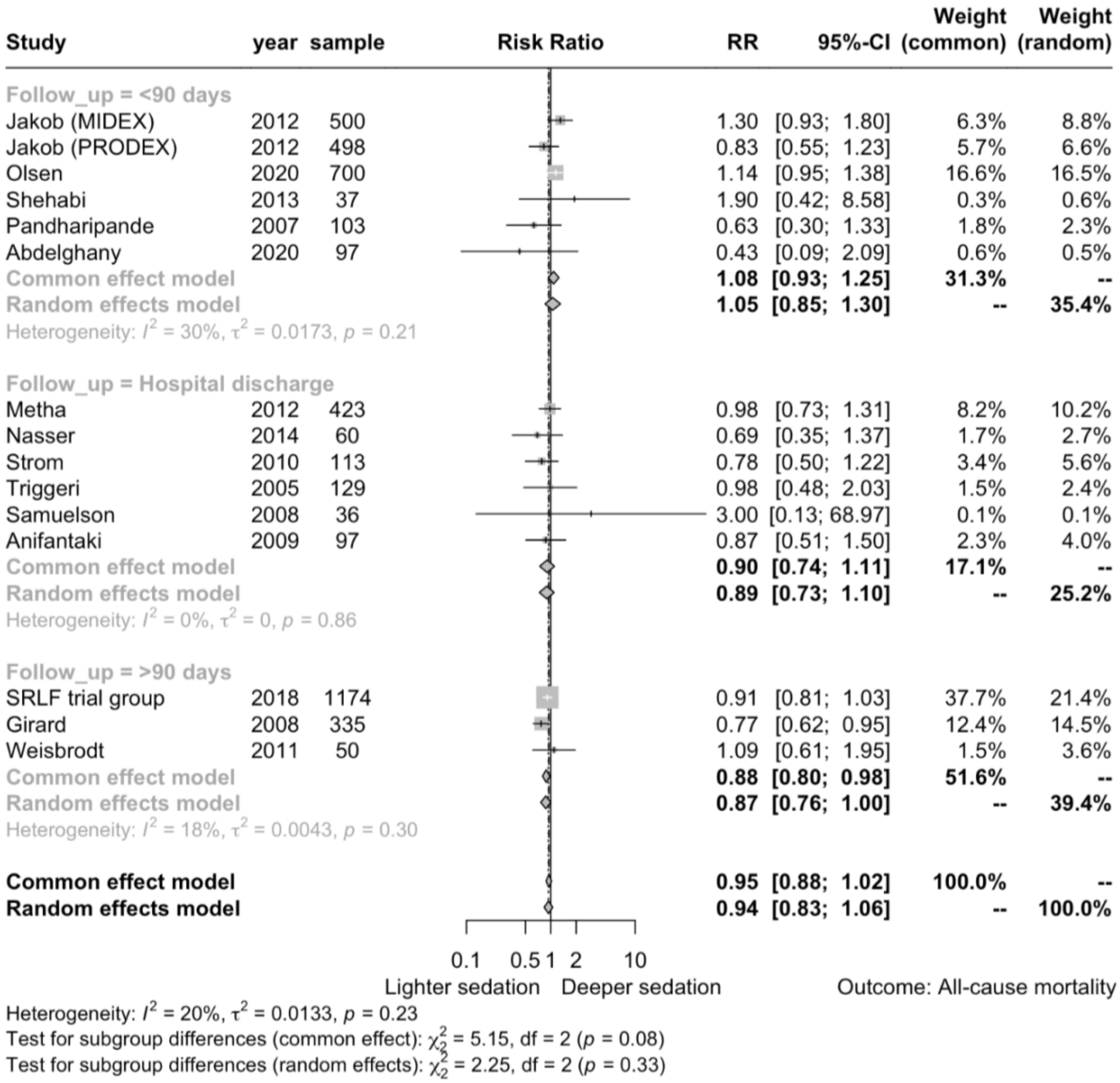

Random effects meta-analysis comparing lighter sedation versus deeper sedation for all-cause mortality with subgroups based on the time to follow-up (risk ratio 0.94, 95% confidence interval 0.83 to 1.06; p-val=0.28; I^2=20%; 15 trials).

Supplement figure 10.

Forest plot for all-cause mortality for all studies in subgroups by the risk of bias.

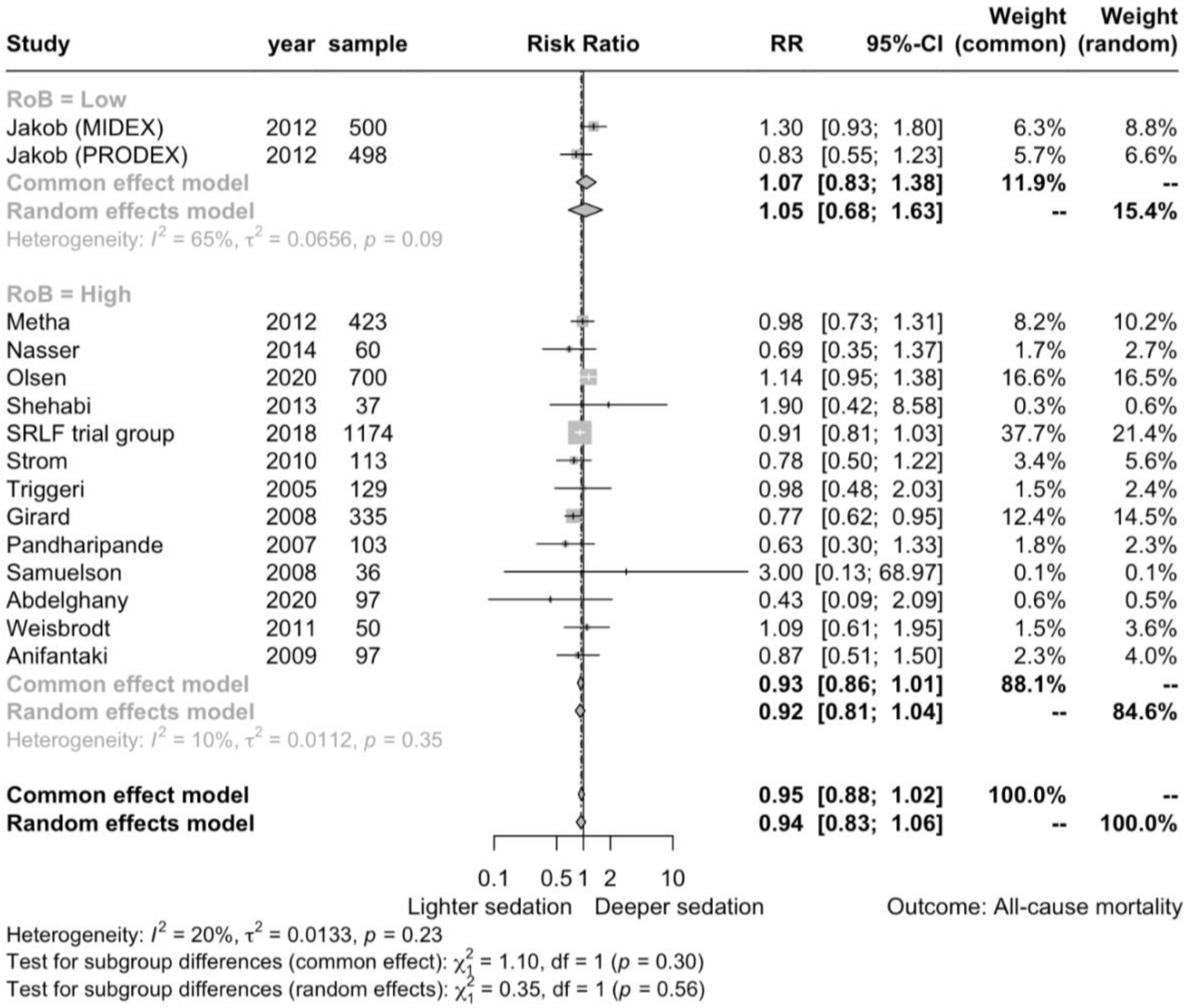

Random effects meta-analysis comparing lighter sedation versus deeper sedation for all-cause mortality with subgroups based on the risk of bias (RoB) (risk ratio 0.94, 95% confidence interval 0.83 to 1.06; p-val=0.28; I^2=20%; 15 trials).

Supplement figure 11.

Forest plot for serious adverse events for all studies in subgroups by intervention type.

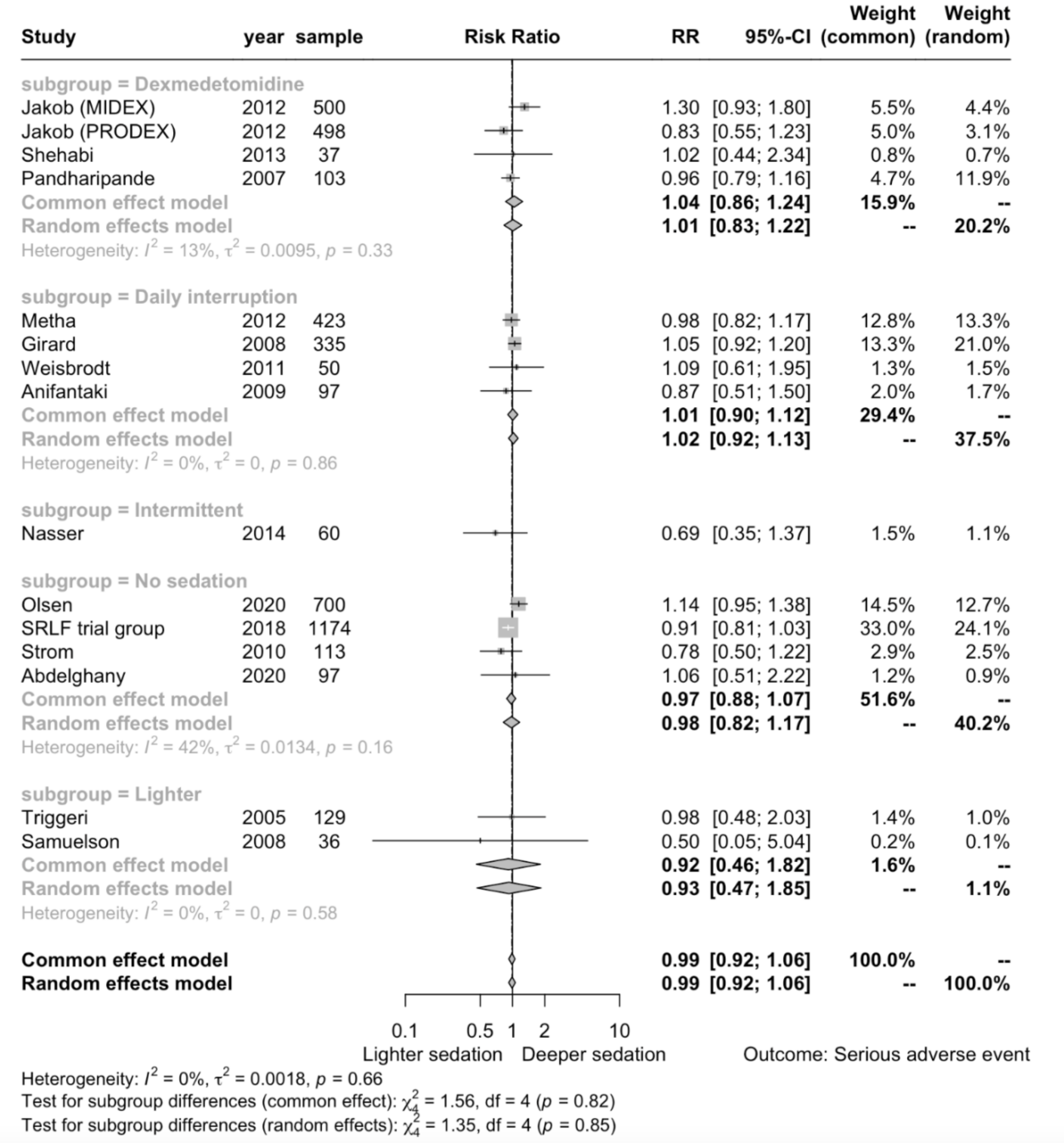

Random effects meta-analysis comparing lighter sedation versus deeper sedation for serious adverse events with subgroups based on intervention type (risk ratio 0.99, 95% confidence interval 0.92 to 1.06; p-val=0.80; I^2=0%; 15 trials).

Supplement figure 12.

Forest plot for serious adverse events for all studies in subgroups by the time to follow up.

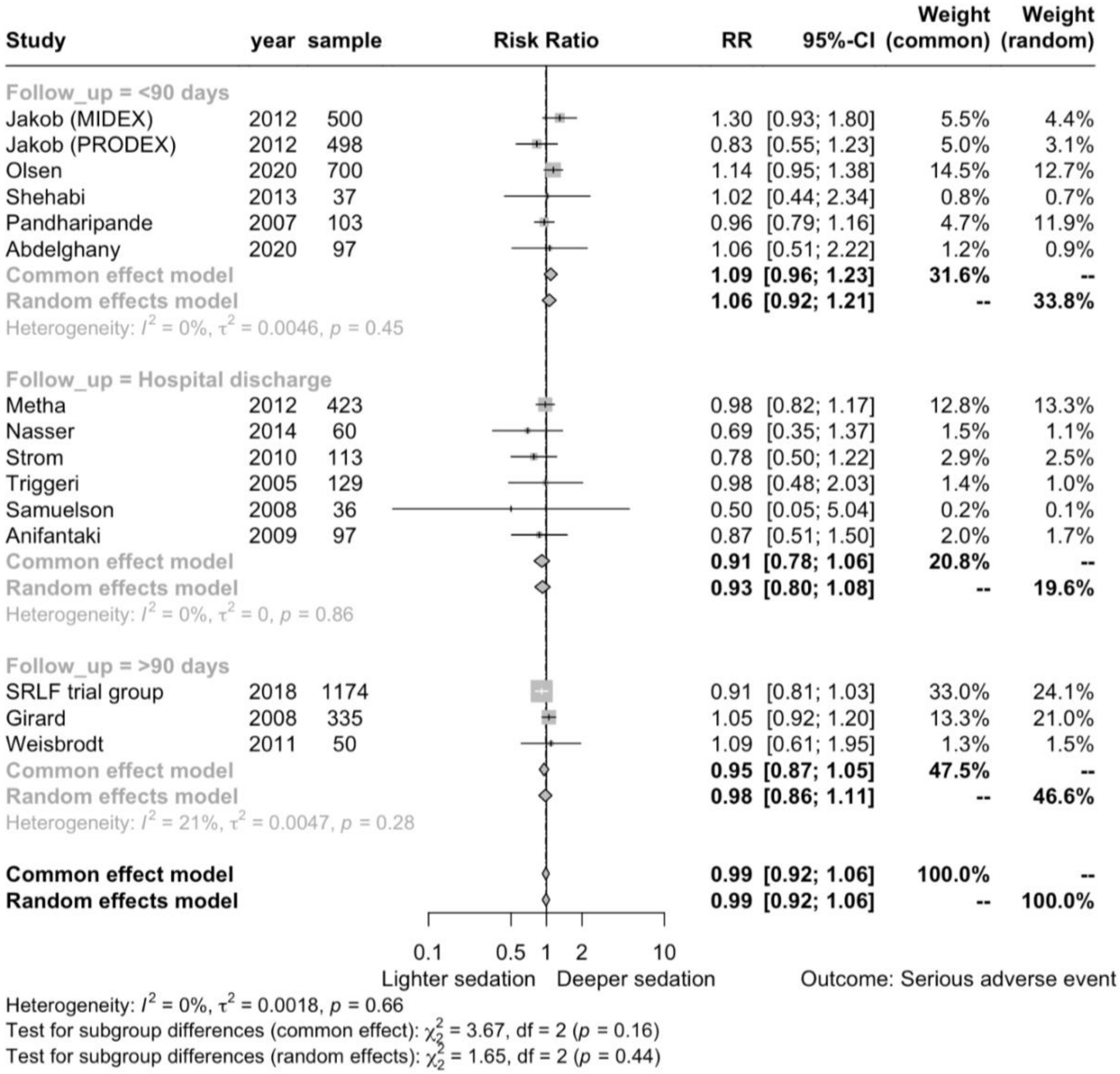

Random effects meta-analysis comparing lighter sedation versus deeper sedation for serious adverse events with subgroups based on the time to follow-up (risk ratio 0.99, 95% confidence interval 0.92 to 1.06; p-val=0.80; I^2=0%; 15 trials).

Supplement figure 13.

Forest plot for serious adverse events for all studies in subgroups by the risk of bias.

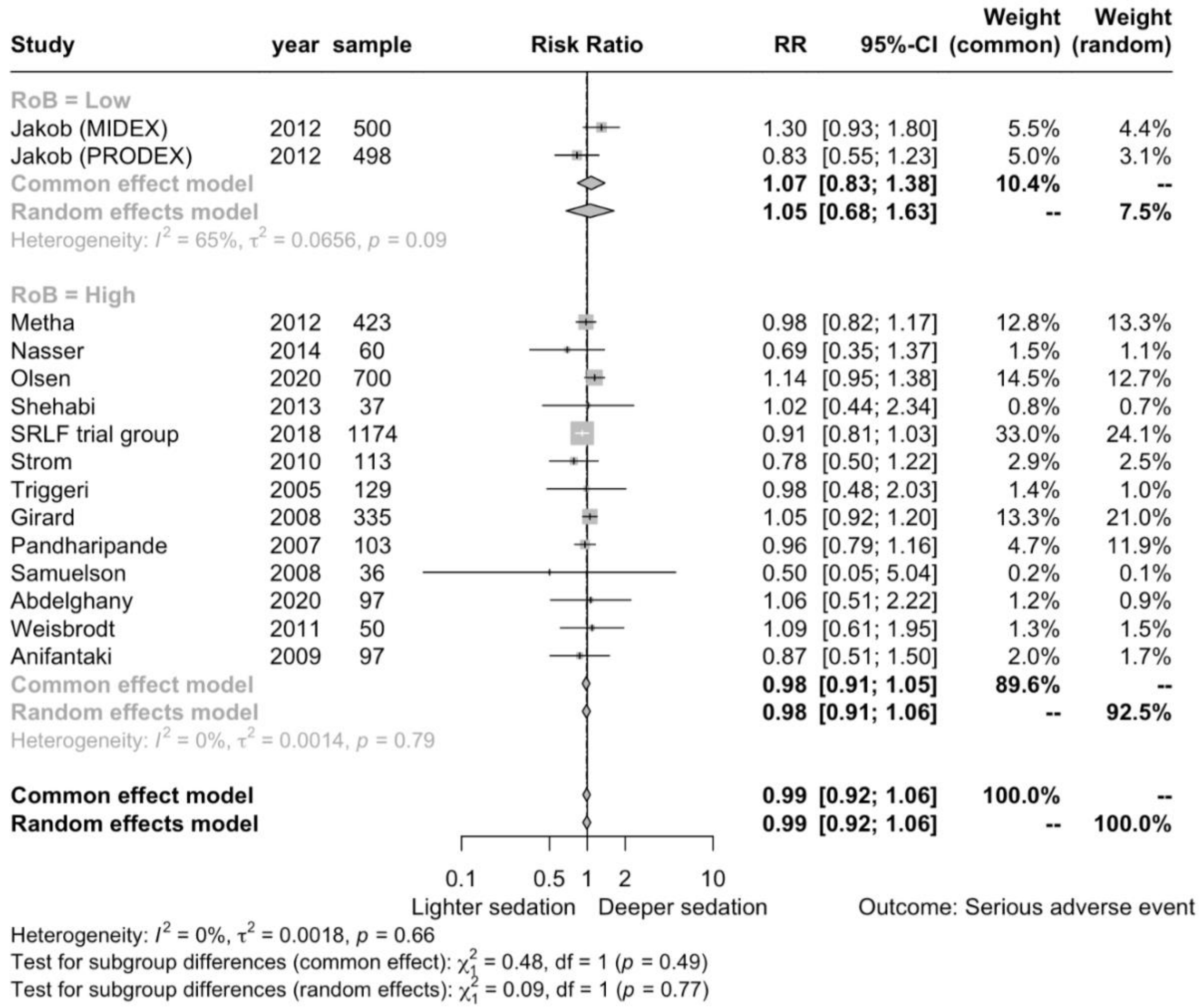

Random effects meta-analysis comparing lighter sedation versus deeper sedation for serious adverse events with subgroups based on the risk of bias (risk ratio 0.99, 95% confidence interval 0.92 to 1.06; p-val=0.28; I^2=0%; 15 trials).

Supplement figure 14.

Forest plot for delirium for all studies in subgroups by the type of intervention.

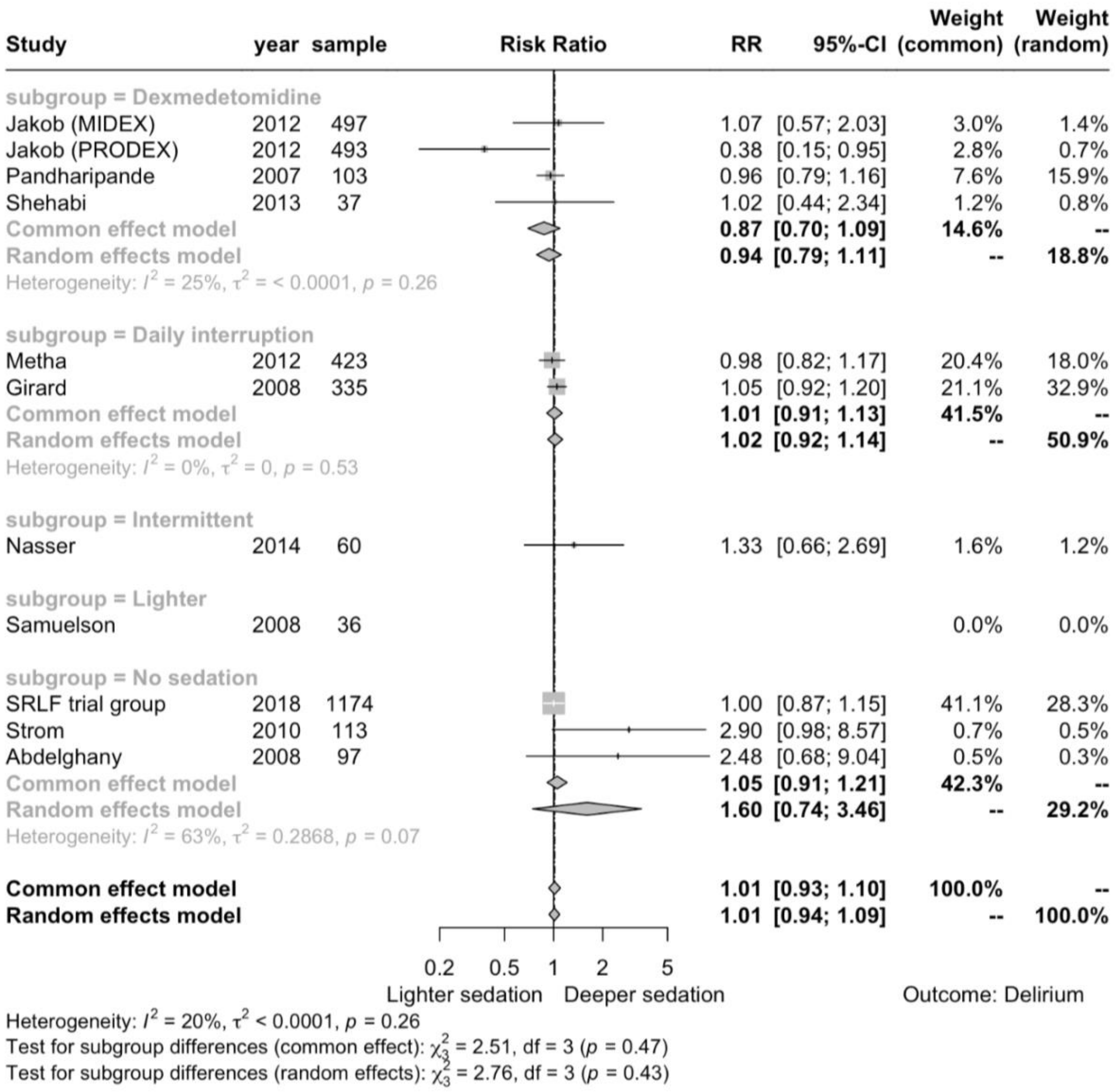

Random effects meta-analysis comparing lighter sedation versus deeper sedation for delirium with subgroups based on intervention type (risk ratio 1.01, 95% confidence interval 0.94 to 1.09; p-val=0.78; I^2=20%; 11 trials).

Supplement figure 15

Forest plot for delirium for all studies in subgroups by the time to follow up.

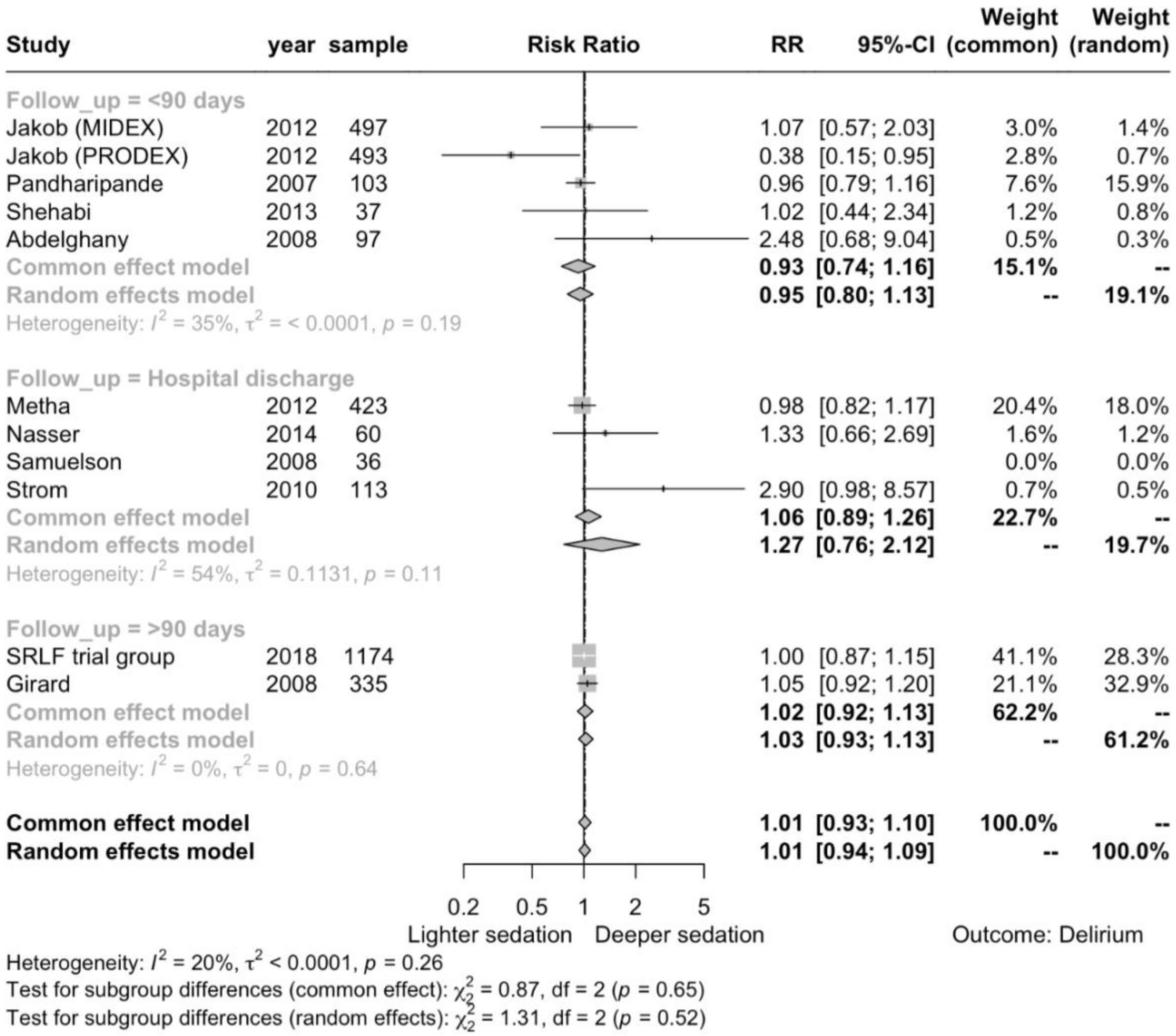

Random effects meta-analysis comparing lighter sedation versus deeper sedation for delirium with subgroups based on the time to follow up (risk ratio 1.01, 95% confidence interval 0.94 to 1.09; p-val=0.78; I^2=20%; 11 trials).

Supplement figure 16

Forest plot for delirium for all studies in subgroups by the risk of bias.

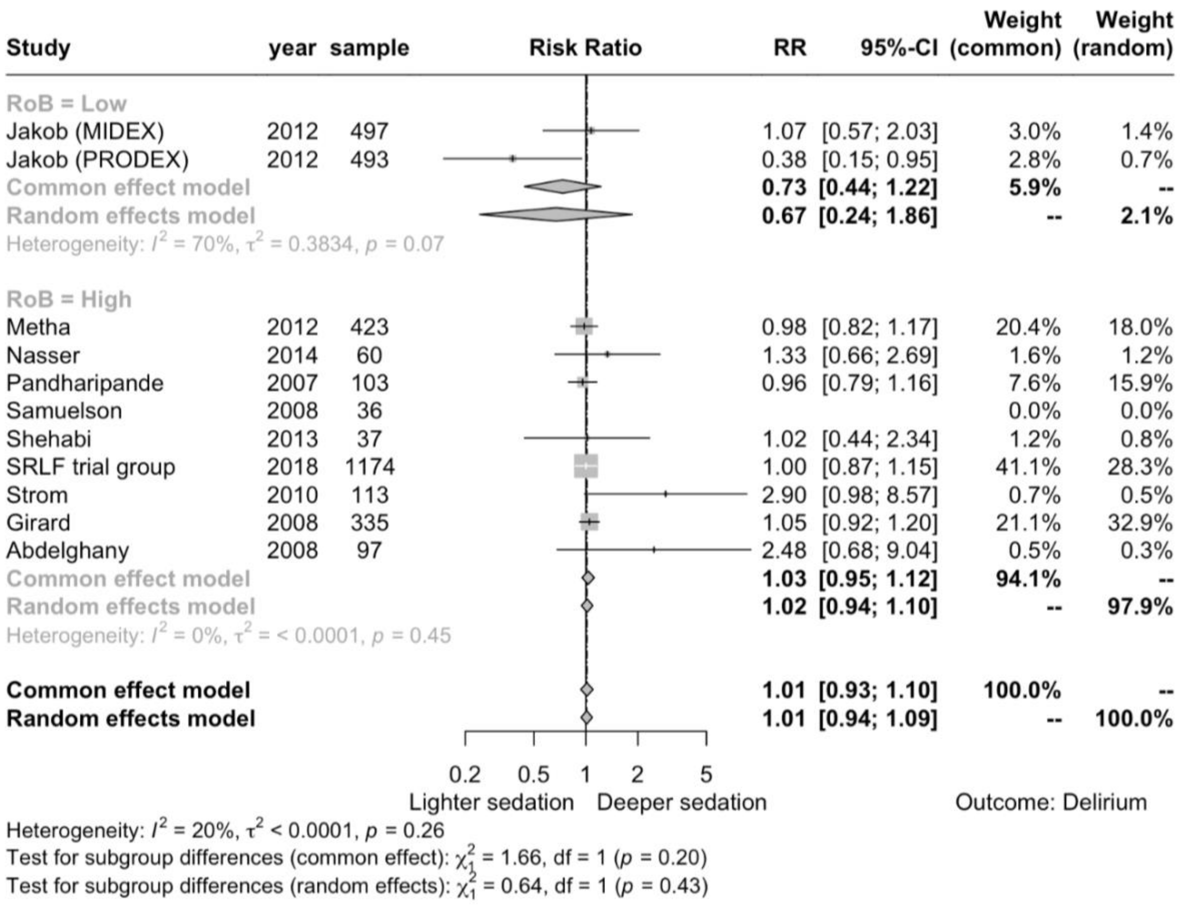

Random effects meta-analysis comparing lighter sedation versus deeper sedation for delirium with subgroups based on the risk of bias (risk ratio 1.01 favoring deeper sedation, 95% confidence interval 0.94 to 1.09; p-val=0.78; I^2=20%; 11 trials).
